# A continuous-time adaptive particle filter for estimations under measurement time uncertainties with an application to a plasma-leucine mixed effects model

**DOI:** 10.1186/1752-0509-7-8

**Published:** 2013-01-19

**Authors:** Annette Krengel, Jan Hauth, Marja-Riitta Taskinen, Martin Adiels, Mats Jirstrand

**Affiliations:** 1 Fraunhofer-Institut für Techno- und Wirtschaftsmathematik (ITWM; Fraunhofer Institute for Industrial Mathematics), Kaiserslautern, Germany; 2 Department of Medicine, University of Helsinki, Helsinki, Finland; 3 Department of Mathematical Sciences, University of Gothenburg, Gothenburg, Sweden; 4 Wallenberg laboratory, Sahlgrenska Center for Cardiovascular Research, Department of Medicine, University of Gothenburg, Gothenburg, Sweden; 5 Fraunhofer Chalmers Centre (FCC), Gothenburg, Sweden

**Keywords:** Particle filter, Sequential Monte Carlo methods, Nonlinear filtering, Parameter estimation, Measurement time uncertainties, PK/PD, Mixed effects, Leucine kinetics

## Abstract

**Background:**

When mathematical modelling is applied to many different application areas, a common task is the estimation of states and parameters based on measurements. With this kind of inference making, uncertainties in the time when the measurements have been taken are often neglected, but especially in applications taken from the life sciences, this kind of errors can considerably influence the estimation results. As an example in the context of personalized medicine, the model-based assessment of the effectiveness of drugs is becoming to play an important role. Systems biology may help here by providing good pharmacokinetic and pharmacodynamic (PK/PD) models. Inference on these systems based on data gained from clinical studies with several patient groups becomes a major challenge. Particle filters are a promising approach to tackle these difficulties but are by itself not ready to handle uncertainties in measurement times.

**Results:**

In this article, we describe a variant of the standard particle filter (PF) algorithm which allows state and parameter estimation with the inclusion of measurement time uncertainties (MTU). The modified particle filter, which we call MTU-PF, also allows the application of an adaptive stepsize choice in the time-continuous case to avoid degeneracy problems. The modification is based on the model assumption of uncertain measurement times. While the assumption of randomness in the measurements themselves is common, the corresponding measurement times are generally taken as deterministic and exactly known. Especially in cases where the data are gained from measurements on blood or tissue samples, a relatively high uncertainty in the true measurement time seems to be a natural assumption. Our method is appropriate in cases where relatively few data are used from a relatively large number of groups or individuals, which introduce mixed effects in the model. This is a typical setting of clinical studies. We demonstrate the method on a small artificial example and apply it to a mixed effects model of plasma-leucine kinetics with data from a clinical study which included 34 patients.

**Conclusions:**

Comparisons of our MTU-PF with the standard PF and with an alternative Maximum Likelihood estimation method on the small artificial example clearly show that the MTU-PF obtains better estimations. Considering the application to the data from the clinical study, the MTU-PF shows a similar performance with respect to the quality of estimated parameters compared with the standard particle filter, but besides that, the MTU algorithm shows to be less prone to degeneration than the standard particle filter.

## Background

### Measurement time uncertainties

Uncertainty in the time at which a measurement is taken is an often neglected source of random error. While in many application areas, this kind of error is generally small and indeed neglectable (due to automated measurements and precise timings), in others it may be of real influence, especially in the life sciences. As a prominent example, one may consider pharmacokinetic and pharmacodynamic (PK/PD) models which are used to describe the metabolic interactions and the effects of a chemical agent (like a drug or a labelled substance) over time inside an organism, respectively.

A typical population experiment in the PK/PD context consists in the analysis of the contents of the blood plasma of several individuals with respect to concentrations of certain molecules of interest. For this purpose, blood probes have to be taken from each individual at certain (fixed) time points after a certain event has occurred (e.g. a drug or a labelled substance has been applied). It is clear from the setting of the experiments that there is some variation in the real point in time when the blood probe has been taken: the true time when the measurement value has been obtained might be shortly before or after the intended time, and this true measurement time is not known to us. Since the inclusion of those time uncertainties in the model usually makes the analysis more difficult, it is standard to lump the time uncertainties with the measurement error. But especially at early times when concentrations change quickly, this may easily lead to wrong estimations, even if one assumes very high variances of the measurement error (we will demonstrate this later on a simple example). On the other hand, the inclusion of measurement time uncertainties (MTU) in algorithms aiming at inference making in complex models is not straightforward. In this article, we will present a modification of the Particle Filter (PF) algorithm (which we call MTU-PF) which is able to fully include a statistical model of the time uncertainties.

### Inference in complex systems

The assessment of the effectiveness of a drug in a clinical study has been done in the past by the direct computation of relatively simple statistical values. The enormous increase in complexity of the underlying models, due to present developments in medicine and biology, for instance in the areas of personalized medicine or systems biology, increases also the need for more sophisticated model-based inference methods.

The estimation of unobservable internal variables or model parameters from data which have been obtained from blood or tissue samples at several time points can reveal information on the concentrations and effectiveness of the substance under question. If these data come from individuals which belong to two different (or even more) groups, e.g. test and control group, mixed effects are introduced in the underlying models. The inherent non-linearity and high variability of biological processes adds considerably to the difficulties one faces during the inference step. Inference in connection with dynamic models plays a major role in many other application areas. State and parameter estimation as well as model discrimination and validation are most common, but also optimal control problems should be mentioned.

It is often not enough to consider (independent) measurement noise [1]. Correlations between residuals are not uncommon, and the violation of this statistical assumption may lead to wrong estimates. A natural way to include correlated noise is to model two different types of noise: the dynamic (process or system) noise which is present in the dynamics of the system states and originates either from true random fluctuations in the system or from unmodelled dynamics in the system, and the measurement noise which is introduced by the measurement procedure or equipment and modelled by independent residuals. One possible approach is to use state space models which consist of a time-continuous model for the system states, e.g. based on Stochastic Differential Equations (SDEs), and a separate model for the time-discrete measurements.

### Parameter estimation with Maximum Likelihood approach

Parameter estimation in state space systems is a difficult problem. In a context where the system dynamics are modelled by Ordinary Differential Equations (ODEs) without correlated noise, the problem is most often considered as a (deterministic) optimization problem based on a Maximum Likelihood (ML) formulation. An overview of these approaches can be found in [2] and [3]; see also [4], which consider also other aspects like identifiability. A generalization of the ML approach including more flexible cost functions is given by the prediction error estimation method ([5]). The introduction of system noise in the state variables leads to optimization problems with SDE constraints. In this case, internal system states which cannot be directly observed need to be estimated jointly with the parameters, given the data. For this purpose, the parameter estimation methods must be augmented by appropriate state filtering methods. An overview of ML parameter estimation in these types of models is given in [6]. If the SDEs are non-linear, linearizations to the Kalman Filter, like the Extended Kalman Filter (EKF) or the Unscented Kalman Filter (UKF), are used to establish approximations to the means and covariances of the filter distributions over time. All those approximations suffer from the fact that they approximate the filtering distributions of the states by a Gaussian distribution at all time points and cannot adequately approximate skewed or multimodal distributions. Better approximations are provided by simulation based methods like Sequential Monte Carlo (SMC) algorithms where good convergence results have been established ([7]). Nevertheless, they suffer from several drawbacks when applied to the joint estimation of dynamic states and fixed parameters ([8-10], see also [11]).

### Parameter estimation in a Bayesian context

In a Bayesian context, in contrast to the “classical” ML approach, a prior distribution is assigned to the parameter vector, hence the parameters can be treated as random variables. In this sense, parameter estimation is done by evaluating the so-called posterior distribution which can be computed (at least theoretically) by Bayes’ theorem given the observations (measurements) and the prior distribution. In the context of high-dimensional spaces, this requires the computation of high-dimensional integrals which is not possible to do analytically. For this purpose, Markov Chain Monte Carlo (MCMC) methods provide powerful tools for the computation of simulation-based approximations to the posterior distribution. Again, in the context of the joint estimation of dynamic states and fixed parameters, the design of good proposal densities is a very difficult problem which renders the use of standard MCMC methods like the Metropolis-Hastings sampler impractical for the purposes of parameter estimation in state space systems.

It has long been a wish to combine both (dynamic) SMC and (static) MCMC methods to provide a general tool for the joint estimation of dynamic states and static parameters. Only recently, Andrieu et al. [11] proposed a very promising combination of both types of Monte Carlo approaches called Particle Markov Chain Monte Carlo (PMCMC) which is generally applicable and where also convergence has been proved.

In the present article, even though the PMCMC approach might be the preferred method for parameter estimation in state space systems, we will concentrate solely on the SMC methods, since our modification affects only this part. However, to be able to do parameter estimation in a pure SMC context, we rely on an approach that is very often used to avoid problems with the estimation of constant parameters. This approach consists in the introduction of artificial dynamics in the parameters, that means the parameters are allowed to slightly change their values over time. In this way, and in a Bayesian context, the parameters can be treated exactly in the same way as the system states. After building an augmented system state by concatenating the parameter vector and the state vector, the joint estimation of states and parameters reduces to filtering of the augmented state vector which makes SMC methods directly applicable to the problem.

### Particle filters for state and parameter estimation

Particle filters ([12-14]) belong to the class of SMC methods for state filtering in state space models. Using the state augmentation approach, the method is also capable of estimating system parameters. The standard particle filter is designed for discrete, non-linear, and non-Gaussian models and can routinely be adapted to the continuous case with measurements at discrete times. The idea of the particle filter is that, at each time point, there is a sample based representation (the weighted particles) of the current estimate of the inner states and parameters which is based on the measurements that have been obtained up to the current time point. The particle cloud is then propagated through time, and the particles and weights are updated accordingly at each time point where measurements are available.

### Non-Linear Mixed Effects models

Estimation in a Non-linear Mixed Effects model (NLME) involves the estimation of both global and individual parameters. With classical maximum likelihood estimation, the individual parameters are random variables equipped with a distribution while the global parameters remain constants with a “true” but unknown value. If the underlying model equations are non-linear, this leads to likelihood functions which are not analytically accessible and one has to rely on approximations. In the context where the system dynamics are modelled by ODEs, the most popular algorithm for NLME parameter estimation in the PK/PD context is the tool NONMEM ([15]). In [1] an estimation algorithm for NLME models based on Stochastic Differential Equations (SDEs) was proposed that uses the First-Order Conditional Estimation (FOCE) method to approximate the likelihood in combination with the EKF estimation in the SDEs. This has been added to NONMEM ([16]). In [17], a comparison between ODE and SDE based parameter estimation has been performed which showed that the interindividual variabilities were in general estimated to be smaller for the SDE model. Donnet and Samson ([18]) proposed a stochastic version of the Expectation-Maximization (SAEM) algorithm (for the estimation of the global parameters) in combination with MCMC methods (for the estimation of states and individual parameters). However, since MCMC exhibits slow mixing properties in the context of the estimation of states and parameters in state space models, in [19] MCMC has been replaced by the more promising PMCMC approach of Andrieu et al. ([11]).

On the other hand, in a Bayesian context, also the global parameters are equipped with a (prior) probability distribution, and the conceptual difference between global and individual parameters vanishes. The mixed effects model can then be considered as a hierarchical model with dependent parameters ([20,21], see also [22] for a more recent population-based Bayesian approach to PK/PD modelling). Simulation-based (Monte Carlo) methods can easily be adapted to this case. Nevertheless, the above mentioned challenges to both SMC and MCMC methods are even higher due to the increased number of states and parameters in NLME models (the number of states and individual parameters has to be multiplied by the number of individuals).

### Aim of the article

Our goal is two-fold: Firstly, we want to show that the particle filter algorithm is applicable (with our modifications) also to more complex models when time uncertainties are formulated explicitly. Secondly, we want to show that the modification may even provide the possibility for further enhancement of the performance of the algorithm by presenting an adaptive time-stepping scheme which is only possible in the context of the new algorithm.

We do not claim that our MTU algorithm generally performs better or worse than the standard filter, nor that it should be the preferred method for estimation in non-linear mixed effects models. Rather, we provide a method which is usable for models where time uncertainties may play a major role. In these cases, it may indeed lead to better estimations. On the other hand, our method transfers the time-discrete particle filter approach, where updates based on the measurements very strictly depend on the measurement times, to a truly time-continuous approach, where updates to the filtering distributions can be performed at every point on the time-scale. Since we want to focus on the time uncertainties, we neglect discussing further issues like identifiability, model evaluation and model discrimination. In our application to the model of plasma-leucine kinetics, we try to avoid these issues by providing ad-hoc values to some of the parameters (especially to the variances of the system states).

### Motivating example

Let us have a look at an example for illustrating the benefits of a separate modelling of measurement time uncertainties. Let us consider a state space system given by the ODE

dq(t)=(-αq(t)+β)dt

with parameters α,β∈R. Here q(t)∈R denotes the state of the system at time *t*. We call the state trajectories obtained by this deterministic system the nominal evolutions of the states. We add noise to the system in a standard way by introducing an additional term $\sigma \;dW_{t}$, with a standard Wiener process ${\left(W_{t}\right)}_{t\in R^{\geq 0}}$ and a diffusion parameter σ∈R. This leads to the following SDE describing the evolution of the state *q* over time:

$$\;dq(t)=(-\alpha q(t)+\beta )\;dt+\sigma \;dW_{t}.$$

Furthermore, let the initial state *q*(0) be given by a log-normal distribution with parameters $\mu_{q_{0}}$ and $\sigma_{q_{0}}^{2}$ (mean and variance of the logarithm of *q*(0), respectively). The parameters chosen in our implementation of this example are shown in Table 1.

**Table 1:** Parameters for the motivating example

	
true value of *α*	1
true value of *β*	3
*σ*	0.05
$\mu_{q_{0}}$	log(1)
$\sigma_{q_{0}}$	0.1
*σ*_*y*_	0.005
distribution of *T*_*j*_	$N({\hat{t}}_{j},0.3^{2})$ truncated at ${\hat{t}}_{j}\pm 1$ and at *t*_0_

**Figure 1:**
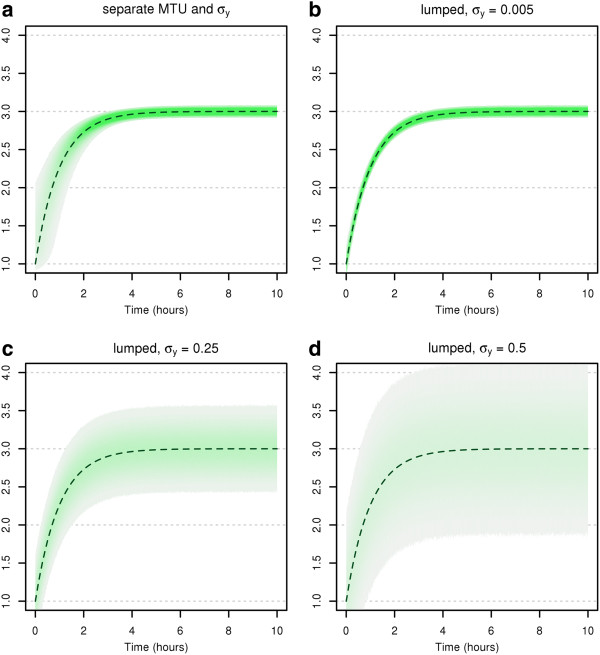
**Assumed measurement distributions for the motivating example.** Measurement distribution resulting from (**a**) separate modelling of measurement time uncertainties and measurement value uncertainties, with *σ*_*y*_=0.005, and (**b**-**d**) lumped time-and-value uncertainties with several different assumed lumped measurement variances *σ*_*y*_. The dashed dark-green line depicts the nominal evolution of the state *q* over time. The green shaded area depicts the region where the measurements are expected.

We assume that *M* measurements of the state *q*(*t*) will be taken at times *T*_*j*_ and that each measurement *j*, *j*=1,…,*M* is disturbed by normal noise with mean *q*(*t*_*j*_) and with a fixed variance $\sigma_{y}^{2}$, i.e. measurement *j* is distributed according to $y_{j}∼N(q(t_{j}),\sigma_{y}^{2})$. Usually, the times *T*_*j*_ are assumed to be known. In contrast, we will assume that in addition to the measurement value error, there is some uncertainty about the exact times where the measurements have been taken. If we attempt to take the *j*th measurement at the intended (or nominal) time ${\hat{t}}_{j}$, the measurement in fact takes place at time *T*_*j*_ which may be shortly before or after the intended time ${\hat{t}}_{j}$. A natural way to model these uncertainties is to assume that the measurement time *T*_*j*_ is given as a realization of a random variable *T*_*j*_. In our example, we assume that *T*_*j*_ follows a truncated normal distribution given by the density

$$\gamma_{j}(t_{j}):=\frac{1}{\Gamma_{j}}\left\{\begin{array}{c} \exp\left(\frac{{\left(t_{j}-{\hat{t}}_{j}\right)}^{2}}{0.3^{2}}\right)\;\text{if}\;\max\{0,{\hat{t}}_{j}-1\}\\ \;\;\;\;\;\;\leq t_{j}\leq {\hat{t}}_{j}+1,\\ 0\;\;\;\;\;\;\;\;\text{otherwise} \end{array}\right)$$

with normalizing constant

$$\Gamma_{j}:=\int_{\max\{0,{\hat{t}}_{j-1}\}}^{{\hat{t}}_{j+1}}\exp\left(\frac{{\left(t_{j}-{\hat{t}}_{j}\right)}^{2}}{0.3^{2}}\right)$$

and given intended measurement times ${\hat{t}}_{j}$. Figure 1 shows the different distributions for one measurement *j* for all possible intended times ${\hat{t}}_{j}$. In each of the subfigures (a)-(d), the shaded green area gives an impression of the “density” of the distribution of the measurement value *y*_*j*_ in dependence of the intended measurement time ${\hat{t}}_{j}$ on the *x*-axis, while the dark-green dashed line depicts the nominal evolution of the state *q* over time. Subfigure (a) shows the distribution of the measurement values with time uncertainties, while (b)-(d) depict the distribution of the measurement values with known measurement times (in this case ${\hat{t}}_{j}=t_{j}$), for several standard deviations *σ*_*y*_: in (b), the original standard deviation is used, while in (c) and (d), higher standard deviations are used which correspond to the cases with lumped value and time variations.

Comparing Figures 1(a) and 1(b-d), we observe that the distributions of the measurements exhibit clearly different shapes. For the “true” model depicted in Figure 1(a), if we consider a single point in time that lies in a time segment where the state values change quickly, the distribution of the measurement at this certain point in time is quite broad. The variance in the measured value is very high, whereas it is small in time segments where the state values change slowly. In contrast, for the standard particle filter, the measurement variance is constant and hence the assumed measurement distributions differ remarkably from the “true” distributions, howsoever the value of $\sigma_{y}^{2}$ is chosen. It must be expected that this leads to difficulties when inference on the states and parameters needs to be done based on these models. We will resume our example after having presented the MTU particle filter and will show that this is indeed the case.

## Methods

We divide this section into three subsections. In the first subsection, we fix the state and observation model we want to consider. In the second subsection entitled “Standard case” we outline the standard particle filter algorithm in the context of time-continuous states with time-discrete measurements, and the various probability distributions involved. Although nothing is new in this subsection, it serves several purposes. Firstly, the time-continuous case is relatively rarely considered in the literature; secondly, the derivation of our modification needs a slightly more general formulation than it is standard for the discrete-time filter; and lastly, the comparison of our modified version with the standard case might more clearly reveal the differences between the two approaches. In the third subsection entitled “MTU particle filter”, we present our new modification of the particle filter. In the following section “Results and Discussion”, we compare the new MTU particle filter to the standard particle filter and to an alternative Maximum Likelihood estimation method on a simple artificial example. We also present an application of our MTU-PF method to a PK/PD study in a non-linear mixed-effects setting in direct comparison with the standard particle filter.

Note: a list of all used symbols with a short explanation can be found at the end of this paper.

### The model

#### State process

Let (Ω,A,P) be a probability space and for each *t*∈ [*t*_0_,*∞*) with $t_{0}\in R$ let $\left(X_{t},B_{X_{t}}\right)$ be an arbitrary measurable space. For each *t*∈ [*t*_0_,*∞*) let further $X_{t}:\Omega \to X_{t}$ be an $A-B_{X_{t}}$ measurable random variable such that $X_{\left[t_{0},\infty \right)}:={\left(X_{t}\right)}_{t\in [t_{0},\infty )}$ is a continuous-time Markov process with general state space

$$X_{\left[t_{0},\infty \right)}:=\underset{t_{0}\leq s}{\prod }X_{s}.$$

For each *t*∈ [*t*_0_,*∞*), denote by $L_{X_{t}}$ the pushforward measure of P under *X*_*t*_, i.e. $L_{X_{t}}(B):\;=\text{P}(X_{t}^{-1}(B))$ for all $B\in B_{X_{t}}$. Further, denote by $L_{X_{\left[t_{0},\infty \right)}}$ the pushforward measure of P under $X_{\left[t_{0},\infty \right)}:\;={\left(X_{s}\right)}_{s\in [t_{0},\infty )}$ (with the corresponding product algebra). Analogously, denote by

$$X_{\left[t_{0},t\right]}:=\underset{t_{0}\leq s\leq t}{\prod }X_{s}\;\text{for each}\;t\geq t_{0}$$

the state space restricted to the interval [*t*_0_,*t*], and denote by $L_{X_{\left[t_{0},t\right]}}$ the corresponding pushforward measure. For each *s* and *t* with *t*>*s*≥*t*_0_, let *K*_*s*,*t*_(*x*_*s*_, d*x*_*t*_) be the Markov kernel of the process $X_{\left[t_{0},\infty \right)}$ from time *s* to time *t*.

An important special case for $X_{\left[t_{0},\infty \right)}$ is given by a multidimensional Itô process on $X_{t}=R^{n}$ (equipped with the corresponding Borel *σ*-algebra) defined through a stochastic differential equation (SDE)

$$\;dX_{t}=a(X_{t},t)\;\;dt+B(X_{t},t)\;\;dW_{t}$$

with drift *a*(*x*,*t*), diffusion matrix *B*(*x*,*t*), multidimensional standard Wiener process $W_{t}$, and initial variable $X_{t_{0}}$. In this case, it is possible to sample directly (at least approximately) from the kernels *K*_*s*,*t*_ when a suitable discretization method is applied, for instance the Euler-Maruyama method.

#### Observations / measurements

Let the process $X_{\left[t_{0},\infty \right)}$ be observed via *M* random variables *Y*_1:*M*_ with values in measurable spaces $\left(Y_{j},B_{Y_{j}}\right)$. Each single observation (measurement) *y*_*j*_ depends on the state variable $x_{t_{j}}$ at some time *T*_*j*_ and on the observation time (measurement time) *T*_*j*_ itself. We assume that, given the observation time *T*_*j*_ and the state $X_{t_{j}}=x_{t_{j}}$, the variable *y*_*j*_ is independent of all other variables, and the conditional measure can be expressed via some conditional probability density $g_{j}(y_{j}\;|\;x_{t_{j}},t_{j})$ with respect to a reference measure $\mu_{Y_{j}}$ on $\left(Y_{j},B_{Y_{j}}\right)$. We do not require any further restrictions on *g* such as linear dependence on the states or Gaussianity.

#### Observation / measurement times

The observation times (measurement times) *T*_*j*_ for *j*=1,…,*M* are usually assumed to be deterministically given and known. Our variant of the particle filter will be based on the assumption that the observation times *T*_*j*_ are themselves realizations of random variables *T*_*j*_. These variables model the uncertainty about exact observation times. In contrast to the observation variables *y*_*j*_, the observation times *T*_*j*_ are never observed (measured). We assume that all information available to us is their probability distribution on the half axis [*t*_0_,*∞*), while in the case of the observations *y*_*j*_, we know both the densities $g_{j}(y_{j}\;|\;x_{t_{j}},t_{j})$*and* the observed values *y*_*j*_.

In this article, we will only consider the simplest case where each variable *T*_*j*_ is independent of all others. Dependencies between the *T*_*j*_’s, especially concerning the order of the observation times, may be considered natural but would lead to more complicated algorithms. However, order dependencies can easily be introduced via restrictions on the support of the variables. In general, the probability distribution of every single variable *T*_*j*_ shall be given by a density *γ*_*j*_(*t*_*j*_) with respect to the Lebesgue measure $\lambda_{\left[t_{0},\infty \right)}$ on the interval [*t*_0_,*∞*).

In the following, we will consider the two cases mentioned, where either all *T*_*j*_ are deterministic and known or all *T*_*j*_ are random and unknown. Note that the first case formally coincides with the case that *T*_*j*_ is random but observed. We will therefore stick to the notation $g_{j}(y_{j}\;|\;x_{t_{j}},t_{j})$ for the observation densities in both cases.

### Standard case: measurement times deterministic and known

We will first consider the standard case, where the observation times *T*_*j*_ are known. For simplicity, we assume here that the observation times *t*_1:*M*_ are strictly ordered increasingly, i.e. *t*_0_<*t*_1_⋯<*t*_*M*_.

The standard case of the particle filter is usually formulated for discrete-time Markov processes $X_{t_{0:M}}:\;=\;{\left(X_{t_{j}}\right)}_{j\in \{0,\ldots ,M\}}$ with general state space where the state variables are only defined at the initial time *t*_0_ and at the times *t*_1_,…,*t*_*M*_ when measurements occur. Nevertheless, this case is included in our more general framework where *X*_*t*_ is defined for all *t*≥*t*_0_. One just focuses on the state variables for those times only. In view of the later generalization to random observation times, we will consider the fixed values *T*_*j*_ as realizations of random variables *T*_*j*_ and condition all occurring densities on them. As mentioned above this assumption leads to the same results as if we assumed the values *T*_*j*_ to be given deterministically.

#### Full model and filter model

The full model is given by the joint density of the variables $X_{t_{0:M}}$ and *Y*_1:*M*_ (conditioned on the observation times *T*_1:*M*_=*t*_1:*M*_) with respect to the product measure $L_{X_{t_{0:M}}}\overset{M}{\underset{j=1}{\prod }}\mu_{Y_{j}}$:

(1) $$f^{X_{t_{0:M}},Y_{1:M}\;|\;T_{1:M}}(x_{t_{0:M}},y_{1:M}\;|\;t_{1:M}):=\overset{M}{\underset{j=1}{\prod }}g_{j}(y_{j}\;|\;x_{t_{j}},t_{j}).$$

The filter at a given time *t*_*k*_ is based on a reduced model. This model is given by the joint density of the variables $X_{t_{0:k}}$ and *Y*_1:*k*_ (conditioned on *T*_1:*M*_=*t*_1:*M*_) with respect to the product measure $L_{X_{t_{0:k}}}\overset{k}{\underset{j=1}{\prod }}\mu_{Y_{j}}$:

(2) $$f^{X_{t_{0:k}},Y_{1:k}\;|\;T_{1:M}}(x_{t_{0:k}},y_{1:k}\;|\;t_{1:M}):=\overset{k}{\underset{j=1}{\prod }}g_{j}(y_{j}\;|\;x_{t_{j}},t_{j}).$$

This density is based on the state sequence $X_{t_{0:k}}$. In contrast, we can focus on the single state $X_{t_{k}}$ by considering the joint density of the variables $X_{t_{k}}$ and *Y*_1:*k*_ (given *T*_1:*M*_=*t*_1:*M*_) with respect to $L_{X_{t_{k}}}\overset{k}{\underset{j=1}{\prod }}\mu_{Y_{j}}$. It can be computed by marginalization as follows:

(3) $$\begin{array}{lll} & f^{X_{t_{k}},Y_{1:k}\;|\;T_{1:M}}(x_{t_{k}},y_{1:k}\;|\;t_{1:M})\; & \;\\ & \;:=\int_{\left\{{\tilde{x}}_{t_{0:k}}\in X_{t_{0:k}}\;:\;{\tilde{x}}_{t_{k}}=x_{t_{k}}\right\}}f^{X_{t_{0:k}},Y_{1:k}\;|\;T_{1:M}}\left({\tilde{x}}_{t_{0:k}},y_{1:k}\;|\;t_{1:M}\right)\; & \;\\ & \;\times dL_{X_{t_{0:k}}}\left({\tilde{x}}_{t_{0:k}}\right)\; \end{array}$$

and the filter density at time *t*_*k*_ with respect to $L_{X_{t_{k}}}$ can then be computed with Bayes’ theorem:

(4) $$\;f^{X_{t_{k}}\;|\;Y_{1:k},T_{1:M}}(x_{t_{k}}\;|\;y_{1:k},t_{1:M}):=\frac{f^{X_{t_{k}},Y_{1:k}\;|\;T_{1:M}}(x_{t_{k}},y_{1:k}\;|\;t_{1:M})}{f^{Y_{1:k}\;|\;T_{1:M}}(y_{1:k}\;|\;t_{1:M})}$$

with

(5) $$\begin{array}{c} f^{Y_{1:k}\;|\;T_{1:M}}(y_{1:k}\;|\;t_{1:M})\\ \;:=\int_{X_{t_{0:k}}}f^{X_{t_{0:k}},Y_{1:k}\;|\;T_{1:M}}\left(x_{t_{0:k}},y_{1:k}\;|\;t_{1:M}\right)\;dL_{X_{t_{0:k}}}\left(x_{t_{0:k}}\right). \end{array}$$

For general (non-linear) models, the practical computation of the filter density is very difficult. Nevertheless, the particle filter computes a Monte Carlo approximation using the fact that the filter densities $f^{X_{t_{k}}\;|\;Y_{1:k},T_{1:M}}$ can be computed recursively. This is done in two steps. We consider the filter distribution at time *t*_*k*-1_ given by the probabilities

(6) $$\begin{array}{c} \text{P}\left(X_{t_{k-1}}\in B\;|\;Y_{1:k-1}=y_{1:k-1},T_{1:M}=t_{1:M}\right)\\ \;=\int_{B}f^{X_{t_{k-1}}\;|\;Y_{1:k-1},T_{1:M}}\left(x_{t_{k-1}}\;|\;y_{1:k-1},t_{1:M}\right)\;dL_{X_{t_{k-1}}}\left(x_{t_{k-1}}\right) \end{array}$$

for each set $B\in B_{X_{t_{k-1}}}$. We then get first the prediction distribution, i.e. the distribution of $X_{t_{k}}$ given the data *Y*_1:*k*-1_ (and *t*_1:*M*_), by use of the kernel $K_{t_{k-1},t_{k}}$:

(7) $$\begin{array}{c} \text{P}(X_{t_{k}}\in B\;|\;Y_{1:k-1}=y_{1:k-1},T_{1:M}=t_{1:M})\\ \;=\int_{B}\int_{X_{t_{k-1}}}f^{X_{t_{k-1}}\;|\;Y_{1:k-1},T_{1:M}}\left(x_{t_{k-1}}\;|\;y_{1:k-1},t_{1:M}\right)\;\;\\ \;\times dL_{X_{t_{k-1}}}\left(x_{t_{k-1}}\right)K_{t_{k-1},t_{k}}\left(x_{t_{k-1}},\;dx_{t_{k}}\right) \end{array}$$

for each set $B\in B_{X_{t_{k}}}$. Then we use Bayes’ theorem to get the filter distribution at time *t*_*k*_:

(8) $$\begin{array}{c} \text{P}(X_{t_{k}}\in B\;|\;Y_{1:k}=y_{1:k},T_{1:M}=t_{1:M})\\ \;=\int_{B}\frac{g_{k}(y_{k}\;|\;x_{t_{k}},t_{k})}{f^{Y_{k}\;|\;Y_{1:k-1},T_{1:M}}(y_{k}\;|\;y_{1:k-1},t_{1:M})}\;\cdot \\ \;\int_{X_{t_{k-1}}}f^{X_{t_{k-1}}\;|\;Y_{1:k-1},T_{1:M}}(x_{t_{k-1}}\;|\;y_{1:k-1},t_{1:M})\;\;\\ \;\;\times dL_{X_{t_{k-1}}}\left(x_{t_{k-1}}\right)K_{t_{k-1},t_{k}}\left(x_{t_{k-1}},\;dx_{t_{k}}\right) \end{array}$$

for each set $B\in B_{X_{t_{k}}}$, with normalizing constant

(9) $$\begin{array}{c} f^{Y_{k}\;|\;Y_{1:k-1},T_{1:M}}(y_{k}\;|\;y_{1:k-1},t_{1:M})\\ \;:=\int_{X_{t_{k}}}g_{k}(y_{k}\;|\;x_{t_{k}},t_{k})\cdot \\ \;\int_{X_{t_{k-1}}}f^{X_{t_{k-1}}\;|\;Y_{1:k-1},T_{1:M}}\left(x_{t_{k-1}}\;|\;y_{1:k-1},t_{1:M}\right)\;\;\\ \;\;\times dL_{X_{t_{k-1}}}\left(x_{t_{k-1}}\right)K_{t_{k-1},t_{k}}\left(x_{t_{k-1}},\;dx_{t_{k}}\right). \end{array}$$

#### Importance sampling

Another ingredient for the particle filter is sequential importance sampling. We assume that a second Markov chain ${\tilde{X}}_{t_{0:M}}$ on the same state space is given with pushforward measures $L_{{\tilde{X}}_{t_{j}}}$ and kernels ${\tilde{K}}_{t_{j-1},t_{j}}\left(x_{t_{j-1}},\;dx_{t_{j}}\right)$ for *j*=1,…,*M*. We assume that for each $x_{t_{j-1}}\in X_{t_{j-1}}$, the measure $K_{t_{j-1},t_{j}}\left(x_{t_{j-1}},\cdot \right)$ is absolutely continuous with respect to the measure ${\tilde{K}}_{t_{j-1},t_{j}}\left(x_{t_{j-1}},\cdot \right)$. It follows that the Radon-Nikodym derivative (written as conditional density)

$$\varrho_{t_{j}\;|\;t_{j-1}}(x_{t_{j}}\;|\;x_{t_{j-1}}):\;=\frac{K_{t_{j-1},t_{j}}(x_{t_{j-1}},\;dx_{t_{j}})}{{\tilde{K}}_{t_{j-1},t_{j}}(x_{t_{j-1}},\;dx_{t_{j}})}$$

exists. We further assume that the pushforward measure $L_{X_{t_{0}}}$ under $X_{t_{0}}$ is absolutely continuous with respect to the corresponding pushforward measure $L_{{\tilde{X}}_{t_{0}}}$ under ${\tilde{X}}_{t_{0}}$ with Radon-Nikodym derivative

$$\varrho_{t_{0}}(x_{t_{0}}):=\frac{\;dL_{X_{t_{0}}}(x_{t_{0}})}{\;dL_{{\tilde{X}}_{t_{0}}}(x_{t_{0}})}.$$

For sequential importance sampling, we need to be able to sample from the initial measure $L_{{\tilde{X}}_{t_{0}}}$ and from the kernels

$${\tilde{K}}_{t_{j-1},t_{j}}\left(x_{t_{j-1}},\cdot \right)$$

for each $x_{t_{j-1}}\in X_{t_{j-1}}$, and to compute $\varrho_{t_{0}}(x_{t_{0}})$ as well as $\varrho_{t_{j}\;|\;t_{j-1}}(x_{t_{j}}\;|\;x_{t_{j-1}})$ pointwise.

Using

$$K_{t_{k-1},t_{k}}\left(x_{t_{k-1}},\;dx_{t_{k}}\right)=\varrho_{t_{k}\;|\;t_{k-1}}\left(x_{t_{k}}\;|\;x_{t_{k-1}}\right){\tilde{K}}_{t_{k-1},t_{k}}\left(x_{t_{k-1}},\;dx_{t_{k}}\right),$$

we can then write the recursive formula (8) for the filter distribution at time *t*_*k*_ as

(10) $$\begin{array}{c} \text{P}(X_{t_{k}}\in B\;|\;Y_{1:k}=y_{1:k},T_{1:M}=t_{1:M})\\ \;=\int_{B}\frac{g_{k}(y_{k}\;|\;x_{t_{k}},t_{k})}{f^{Y_{k}\;|\;Y_{1:k-1},T_{1:M}}(y_{k}\;|\;y_{1:k-1},t_{1:M})}\cdot \\ \;\int_{X_{t_{k-1}}}f^{X_{t_{k-1}}\;|\;Y_{1:k-1},T_{1:M}}\left(x_{t_{k-1}}|y_{1:k-1},t_{1:M}\right)\varrho_{t_{k}\;|\;t_{k-1}}\left(x_{t_{k}}|x_{t_{k-1}}\right)\\ \;\times dL_{X_{t_{k-1}}}\left(x_{t_{k-1}}\right){\tilde{K}}_{t_{k-1},t_{k}}(x_{t_{k-1}},\;dx_{t_{k}}) \end{array}$$

for each $B\in B_{X_{t_{k}}}$. The direct computation of the normalizing constants $f^{Y_{k}\;|\;Y_{1:k-1},T_{1:M}}(y_{k}\;|\;y_{1:k-1},t_{1:M})$ (while *Y*_1:*M*_ is assumed to be fixed) is not necessary. Sequential importance sampling is performed as follows. Draw a number *N* of realizations $x_{t_{0}}^{i}$ from $L_{{\tilde{X}}_{t_{0}}}$ and compute the corresponding unnormalized weights

$$w_{t_{0}}^{i}:=\varrho_{t_{0}}\left(x_{t_{0}}^{i}\right)\;\text{for all}\;i=1,\ldots ,N.$$

Then, for all *k*=1,…,*M*, sample realizations $x_{t_{k}}^{i}$ from the kernel ${\tilde{K}}_{t_{k-1},t_{k}}(x_{t_{k-1}}^{i},\;dx_{t_{k}})$ for each *i*=1,…,*N* and compute the unnormalized weights

$$\begin{array}{l} w_{t_{k}}^{i}:=\varrho_{t_{k}\;|\;t_{k-1}}\left(x_{t_{k}}^{i}\;|\;x_{t_{k-1}}^{i}\right)g_{k}\left(y_{k}\;|\;x_{t_{k}}^{i},t_{k}\right)w_{t_{k-1}}^{i}\;\text{for all}\\ \;i=1,\ldots ,N. \end{array}$$

For suitable integrable functions *h* (e.g. fulfilling some mild restrictions on how fast *h* may increase with *x*, see [23] for details), the expectation of *h* with respect to the filter density conditioned on the observations *Y*_1:*k*_=*y*_1:*k*_, given by

(11) $$\begin{array}{c} \text{E}\left[h\left(X_{t_{k}}\right)\;|\;Y_{1:k}=y_{1:k},T_{1:M}=t_{1:M}\right]\\ :={\text{E}}_{f^{X_{t_{k}}\;|\;Y_{1:k}=y_{1:k},T_{1:M}=t_{1:M}}(\cdot \;|\;y_{1:k},t_{1:M})}\left[h(X_{t_{k}})\right]\\ \;=\int f^{X_{t_{k}}\;|\;Y_{1:k},T_{1:M}}(x_{t_{k}}\;|\;y_{1:k},t_{1:M})h(x_{t_{k}})dL_{X_{t_{k}}}\left(x_{t_{k}}\right), \end{array}$$

can then be approximated by

(12) $$\begin{array}{c} {\text{E}}_{t_{k},N}\left[h\left(X_{t_{k}}\right)\;|\;Y_{1:k}=y_{1:k},T_{1:M}=t_{1:M}\right]:=\frac{\overset{N}{\underset{i=1}{\sum }}w_{t_{k}}^{i}h\left(x_{t_{k}}^{i}\right)}{\overset{N}{\underset{i=1}{\sum }}w_{t_{k}}^{i}} \end{array}$$

where *N* is the number of particles. In fact, it can be shown that as *N* approaches infinity, these empirical expectations converge to the filter expectations:

(13) $$\begin{array}{c} \underset{N\to \infty }{\lim}{\text{E}}_{t_{k},N}\left[h(X_{t_{k}})\;|\;Y_{1:k}=y_{1:k},T_{1:M}=t_{1:M}\right]\\ \;=\text{E}\left[h\left(X_{t_{k}}\right)\;|\;Y_{1:k}=y_{1:k},T_{1:M}=t_{1:M}\right]. \end{array}$$

Note that if we can sample from the Markov kernels of $x_{t_{j}}$, we can choose ${\tilde{X}}_{t_{j}}=X_{t_{j}}$ (at least in law), whence $\varrho_{t_{0}}(x_{t_{0}})\equiv 1$ and $\varrho_{t_{j}\;|\;t_{j-1}}(x_{t_{j}}\;|\;x_{t_{j-1}})\equiv 1$. This is a standard choice, but in terms of efficiency of the particle filter algorithm not always the best one. On the other hand, finding a suitable Markov chain ${\tilde{X}}_{t_{0:M}}$ different from $X_{t_{0:M}}$ is not an easy task.

#### Resampling

If the number *N* of samples through time is fixed, the samples obtained by sequential importance sampling quickly degenerate since most of the normalized weights decrease rapidly towards 0. The degree of degeneracy is often measured by an estimate of the so-called effective sample size (ESS). This estimate at time *t* is given by

(14) $$n_{\text{ESS}}:=\frac{1}{\overset{N}{\underset{i=1}{\sum }}{\left({\tilde{w}}_{t}^{i}\right)}^{2}}$$

where

(15) $${\tilde{w}}_{t}^{i}:=\frac{w_{t}^{i}}{\overset{N}{\underset{\nu =1}{\sum }}w_{t}^{\nu }}$$

are the normalized weights. It obtains its maximal value *N* if all weights are equal, and it approaches 1 if the variance of the weights and thus the degree of degeneracy increases. To avoid this degeneration of the samples, a resampling step needs to be done when the ESS drops below a threshold *N*_Threshold_ (which is usually chosen to be *N*/2).

Resampling at some time *s*_*ℓ*_ is based on given non-negative (unnormalized) selection weights $v_{s_{\ell }}^{i}$ for each particle index *i*: One repeatedly selects particles with probabilities $p_{\ell }^{i}$ given by the normalized selection weights

(16) $$p_{\ell }^{i}:=\frac{v_{s_{\ell }}^{i}}{\overset{N}{\underset{\nu =1}{\sum }}v_{s_{\ell }}^{\nu }}.$$

This is multinomial resampling. There exist procedures where each single particle is still selected with probability $p_{\ell }^{i}$, but with reduced overall variance, for instance stratified resampling or systematic resampling which should be preferred [24,25]. In any case, resampling defines a selection function *ι*_*ℓ*_:*I*→*I* on the index set *I*: ={1,…,*N*}. Resampling is then done by replacing the state samples ${\left(x_{s_{\ell }}^{i}\right)}_{i=1,\ldots ,N}$ by the selected state samples ${\left(x_{s_{\ell }}^{\iota_{\ell }(i)}\right)}_{i=1,\ldots ,N}$. Since before selection the probability that the particle *i* will be chosen is $p_{\ell }^{i}$ for each draw, the expected number of times that particle *i* has been chosen after *N* draws is $Np_{\ell }^{\iota_{\ell }(i)}$. To correct for the introduced bias, the normalized weight ${\tilde{w}}_{s_{\ell }}^{i}$ for each selected particle *i* needs then to be corrected by replacing it by the weight

(17) $$\frac{{\tilde{w}}_{s_{\ell }}^{\iota_{\ell }(i)}}{Np_{\ell }^{\iota_{\ell }(i)}}/\overset{N}{\underset{\nu =1}{\sum }}\frac{{\tilde{w}}_{s_{\ell }}^{\iota_{\ell }(\nu )}}{Np_{\ell }^{\iota_{\ell }(\nu )}}=\frac{w_{s_{\ell }}^{\iota_{\ell }(i)}}{v_{s_{\ell }}^{\iota_{\ell }(i)}}/\overset{N}{\underset{\nu =1}{\sum }}\frac{w_{s_{\ell }}^{\iota_{\ell }(\nu )}}{v_{s_{\ell }}^{\iota_{\ell }(\nu )}}$$

(using (16)). The necessary correction is therefore achieved if the unnormalized weights ${\left(w_{s_{\ell }}^{i}\right)}_{i=1,\ldots ,N}$ are replaced by the corrected unnormalized weights ${\left(w_{s_{\ell }}^{\iota_{\ell }(i)}/v_{s_{\ell }}^{\iota_{\ell }(i)}\right)}_{i=1,\ldots ,N}$.

Note that in the original particle filter, the selection weights $v_{s_{\ell }}^{i}$ at time *s*_*ℓ*_ are chosen to be the particle weights (before the replacement), i.e.

$$v_{s_{\ell }}^{i}=w_{s_{\ell }}^{i}\;\text{for}\;i=1,\ldots ,N,$$

such that after the resampling step the unnormalized weights are all equal to 1. Nevertheless, in general their choice is free and may be based on the observations (which is used in the so-called auxiliary particle filter [26]).

#### Particle filter algorithm

The particle filter computes the state realizations and weights recursively through time. In its standard form, the particle filter can be stated as in algorithm 1.

Note that if one chooses ${\tilde{X}}_{\left[t_{0},\infty \right)}=X_{\left[t_{0},\infty \right)}$ (in law), then $\varrho_{t_{k}\;|\;t_{k-1}}(x_{t_{k}}^{i}\;|\;x_{t_{k-1}}^{i})\equiv 1$ and the update of the weights simplifies to

$$w_{t_{k}}^{i}=g_{k}\left(y_{k}\;|\;x_{t_{k}}^{i},t_{k}\right)w_{t_{k-1}}^{i}.$$

#### Data Likelihood

Model validation or discrimination is generally based on the data likelihood

(18) $$\begin{array}{c} Z_{t_{k}}(t_{1:M}):=f^{Y_{1:k}\;|\;T_{1:M}}(y_{1:k}\;|\;t_{1:M})\\ \;=\underset{X_{t_{0:k}}}{\int }f^{X_{t_{0:k}},Y_{1:k}\;|\;T_{1:M}}\left(x_{t_{0:k}},y_{1:k}\;|\;t_{1:M}\right)\;\;dL_{X_{t_{0:k}}}\left(x_{t_{0:k}}\right)\\ \;=\text{E}\left[f^{X_{t_{0:k}},Y_{1:k}\;|\;T_{1:M}}(\;\cdot \;,y_{1:k}\;|\;t_{1:M})\right] \end{array}$$

##### Algorithm 1 Standard particle filter

for given observations *Y*_1:*k*_. Without resampling, the data likelihood could be approximated by the empirical mean of the unnormalized weights, i.e. by

(19) $${\hat{Z}}_{t_{k}}(t_{1:M}):=\frac{1}{N}\overset{N}{\underset{i=1}{\sum }}w_{t_{k}}^{i}$$

because this is the empirical estimate for the above expectation. After a resampling step, this is not valid any longer. Nevertheless, in any case, the data likelihood can be computed recursively by the following estimate of the ratio $Z_{t_{k}}(t_{1:M})/Z_{t_{k-1}}(t_{1:M})$:

(20) $$\begin{array}{c} \frac{\hat{Z_{t_{k}}(t_{1:M})}}{Z_{t_{k-1}}(t_{1:M})}:=\frac{\overset{N}{\underset{i=1}{\sum }}\varrho_{t_{k}\;|\;t_{k-1}}\left(x_{t_{k}}^{i}\;|\;x_{t_{k-1}}^{i}\right)g_{k}\left(y_{k}\;|\;x_{t_{k}}^{i},t_{k}\right)w_{t_{k-1}}^{i}}{\overset{N}{\underset{i=1}{\sum }}w_{t_{k-1}}^{i}}, \end{array}$$

with initial estimate ${\hat{Z}}_{t_{0}}(t_{1:M})=1$ (see e.g. [27]).

### MTU particle filter: Uncertain measurement times

We now assume that each observation time *T*_*j*_ is a realization of a random variable *T*_*j*_. Its distribution is expressed via densities *γ*_*j*_ with respect to the Lebesgue measure $\lambda_{\left[t_{0},\infty \right)}$. The observation times *T*_*j*_ themselves are not observed.

#### Full model

The full model in this case will include complete continuous state paths, since the observation times are now distributed over the complete time axis [*t*_0_,*∞*), and the observations may potentially depend on every state $x_{t_{j}}$ for *t*_*j*_∈[*t*_0_,*∞*). Consider therefore the joint density of the variables $X_{\left[t_{0},\infty \right)}$, *Y*_1:*M*_ and *t*_1:*M*_, with respect to the product measure $L_{X_{\left[t_{0},\infty \right)}}\overset{M}{\underset{j=1}{\prod }}\mu_{Y_{j}}\overset{M}{\underset{j=1}{\prod }}\lambda_{\left[t_{0},\infty \right)}$:

(21) $$\begin{array}{c} f^{X_{\left[t_{0},\infty \right)},Y_{1:M},T_{1:M}}\left(x_{\left[t_{0},\infty \right)},y_{1:M},t_{1:M}\right)=\overset{M}{\underset{j=1}{\prod }}g_{j}(y_{j}\;|\;x_{t_{j}},t_{j})\overset{M}{\underset{j=1}{\prod }}\gamma_{j}(t_{j}). \end{array}$$

#### Filter model

The filter at a given time *t*≥*t*_0_ is again based on a reduced model. This model is given by the joint density of the following variables: $x_{\left[t_{0},t\right]}$, denoting the state paths until time *t*; further only those variables *y*_*j*_ for which *T*_*j*_≤*t*; and finally *t*_1:*M*_. This density is given with respect to the product measure $L_{X_{\left[t_{0},t\right]}}\overset{M}{\underset{j=1}{\prod }}\mu_{Y_{j}}\overset{M}{\underset{j=1}{\prod }}\lambda_{\left[t_{0},\infty \right)}$ by:

(22) $$\begin{array}{c} {\hat{f}}_{t}^{X_{\left[t_{0},t\right]},Y_{1:M},T_{1:M}}(x_{\left[t_{0},t\right]},y_{1:M},t_{1:M})=\overset{M}{\underset{\begin{array}{c} j=1\\ t_{j}\leq t \end{array}}{\prod }}g_{j}(y_{j}\;|\;x_{t_{j}},t_{j})\overset{M}{\underset{j=1}{\prod }}\gamma_{j}(t_{j}). \end{array}$$

Note that we cannot use the simple notation of the standard case where for filtering only the first *k* observations are taken into consideration at time *t*_*k*_, since neither the observations are ordered in time nor the times *T*_*j*_ are fixed in advance. For this reason we have to include all measurements *Y*_1:*M*_ also into the filter model. Note that even though we use the complete data *Y*_1:*M*_=*y*_1:*M*_ in the notation, only those *y*_*j*_ have to be known at time *t* for which *T*_*j*_≤*t* holds. To avoid confusion, we mark all densities connected to the filter model at time *t* by a hat superscript (and by the index *t*).

We will now derive formulas for the filter density. Since we assume that the observation times *t*_1:*M*_ are not observed, we use marginalization to get the joint density for $x_{\left[t_{0},t\right]}$ and *Y*_1:*M*_ only, which is

(23) $$\begin{array}{c} {\hat{f}}_{t}^{X_{\left[t_{0},t\right]},Y_{1:M}}(x_{\left[t_{0},t\right]},y_{1:M})\\ \;=\int_{t_{0}}^{\infty }\cdots \int_{t_{0}}^{\infty }{\hat{f}}_{t}^{X_{\left[t_{0},t\right]},Y_{1:M},T_{1:M}}(x_{\left[t_{0},t\right]},y_{1:M},t_{1:M})\;\;dt_{1}\cdots \;dt_{M}\\ \;=\int_{t_{0}}^{\infty }\cdots \int_{t_{0}}^{\infty }\overset{M}{\underset{\begin{array}{c} j=1\\ t_{j}\leq t \end{array}}{\prod }}g_{j}(y_{j}\;|\;x_{t_{j}},t_{j})\overset{M}{\underset{j=1}{\prod }}\gamma_{j}(t_{j})\;\;dt_{1}\cdots \;dt_{M} \end{array}$$

with respect to the product measure $L_{X_{\left[t_{0},t\right]}}\overset{M}{\underset{j=1}{\prod }}\mu_{Y_{j}}$. We will further simplify this density. If we define

$${\bar{g}}_{j,t}\left(y_{j}\;|\;x_{t_{j}},t_{j}\right):=\left\{\begin{array}{c} g_{j}(y_{j}\;|\;x_{t_{j}},t_{j})\;\text{if}\;t_{j}\leq t\\ 1\;\;\;\;\text{otherwise}, \end{array}\right)$$

then

$$\overset{M}{\underset{\begin{array}{c} j=1\\ t_{j}\leq t \end{array}}{\prod }}g_{j}\left(y_{j}\;|\;x_{t_{j}},t_{j}\right)=\overset{M}{\underset{j=1}{\prod }}{\bar{g}}_{j,t}\left(y_{j}\;|\;x_{t_{j}},t_{j}\right)$$

and further

(24) $$\begin{array}{c} {\hat{f}}_{t}^{X_{\left[t_{0},t\right]},Y_{1:M}}(x_{\left[t_{0},t\right]},y_{1:M})\\ \;=\int_{t_{0}}^{\infty }\cdots \int_{t_{0}}^{\infty }\overset{M}{\underset{\begin{array}{c} j=1\\ t_{j}\leq t \end{array}}{\prod }}g_{j}\left(y_{j}\;|\;x_{t_{j}},t_{j}\right)\overset{M}{\underset{j=1}{\prod }}\gamma_{j}(t_{j})\;\;dt_{1}\cdots \;dt_{M}\\ \;=\int_{t_{0}}^{\infty }\cdots \int_{t_{0}}^{\infty }\overset{M}{\underset{j=1}{\prod }}\left({\bar{g}}_{j,t}\left(y_{j}\;|\;x_{t_{j}},t_{j}\right)\gamma_{j}(t_{j})\right)\;dt_{1}\cdots \;dt_{M}\\ \;=\overset{M}{\underset{j=1}{\prod }}\int_{t_{0}}^{\infty }{\bar{g}}_{j,t}(y_{j}\;|\;x_{t_{j}},t_{j})\gamma_{j}(t_{j})\;dt_{j}, \end{array}$$

where the last step is possible because the factor indexed by *j* does not depend on $t_{j^{\prime }}$ for *j*^′^≠*j*. For each *j*, we can split the integration by *T*_*j*_ at the time point *t* into two parts and get

$$\begin{array}{c} \int_{t_{0}}^{\infty }{\bar{g}}_{j,t}\left(y_{j}\;|\;x_{t_{j}},t_{j}\right)\gamma_{j}(t_{j})\\ \;=\int_{t_{0}}^{t}g_{j}\left(y_{j}\;|\;x_{t_{j}},t_{j}\right)\gamma_{j}(t_{j})\;\;dt_{j}+\int_{t}^{\infty }\gamma_{j}(t_{j})\;\;dt_{j}\\ \;=1+\int_{t_{0}}^{t}\left(g_{j}(y_{j}\;|\;x_{t_{j}},t_{j})-1\right)\gamma_{j}(t_{j})\;\;dt_{j} \end{array}$$

where the last step follows from the fact that *γ*_*j*_ is a probability density and therefore

$$\int_{t_{0}}^{t}\gamma_{j}(t_{j})\;\;dt_{j}+\int_{t}^{\infty }\gamma_{j}(t_{j})\;\;dt_{j}=\int_{t_{0}}^{\infty }\gamma_{j}(t_{j})\;\;dt_{j}=1$$

holds. Inserting this into (24), we get

$$\begin{array}{c} {\hat{f}}_{t}^{X_{\left[t_{0},t\right]},Y_{1:M}}\left(x_{\left[t_{0},t\right]},y_{1:M}\right)\\ \;=\overset{M}{\underset{j=1}{\prod }}\left(1+\int_{t_{0}}^{t}\left(g_{j}(y_{j}\;|\;x_{t_{j}},t_{j})-1\right)\gamma_{j}(t_{j})\;dt_{j}\right). \end{array}$$

With a further marginalization, we get the joint density of *X*_*t*_ and *Y*_1:*M*_ for the filter model,

(25) $$\begin{array}{c} {\hat{f}}_{t}^{X_{t},Y_{1:M}}(x_{t},y_{1:M})\\ \;:=\int_{\left\{{\tilde{x}}_{\left[t_{0},t\right]}\in X_{\left[t_{0},t\right]}\;:\;{\tilde{x}}_{t}=x_{t}\right\}}{\hat{f}}_{t}^{X_{\left[t_{0},t\right]},Y_{1:M}}({\tilde{x}}_{\left[t_{0},t\right]},y_{1:M})\;\;dL_{X_{\left[t_{0},t\right]}}({\tilde{x}}_{\left[t_{0},t\right]})\\ \;=\int_{\left\{{\tilde{x}}_{\left[t_{0},t\right]}\in X_{\left[t_{0},t\right]}\;:\;{\tilde{x}}_{t}=x_{t}\right\}}\\ \;\;\;\overset{M}{\underset{j=1}{\prod }}\left(1+\int_{t_{0}}^{t}\left(g_{j}(y_{j}\;|\;{\tilde{x}}_{t_{j}},t_{j})-1\right)\gamma_{j}(t_{j})\;\;dt_{j}\right)\;\;dL_{X_{\left[t_{0},t\right]}}({\tilde{x}}_{\left[t_{0},t\right]}) \end{array}$$

which is with respect to the product measure $L_{X_{t}}\overset{M}{\underset{j=1}{\prod }}\mu_{Y_{j}}$. From this density, we finally can compute the filter density with respect to $L_{X_{t}}$ by applying Bayes’ theorem:

(26) $${\hat{f}}_{t}^{X_{t}\;|\;Y_{1:M}}(x_{t}\;|\;y_{1:M})=\frac{{\hat{f}}_{t}^{X_{t},Y_{1:M}}(x_{t},y_{1:M})}{{\hat{f}}_{t}^{Y_{1:M}}(y_{1:M})}$$

where

(27) $${\hat{f}}_{t}^{Y_{1:M}}(y_{1:M})=\int_{X_{t}}{\hat{f}}_{t}^{X_{t},Y_{1:M}}({\tilde{x}}_{t},y_{1:M})\;\;dL_{X_{t}}({\tilde{x}}_{t})$$

is the data likelihood with respect to the measure $\overset{M}{\underset{j=1}{\prod }}\mu_{Y_{j}}$.

#### Effective computation of the filter distributions

In the following paragraph, we will show how the densities of the filter distributions given by (26) can be effectively computed. This is the basis for the formulation of our MTU particle filter method.

Let the observations $y_{1:M}\in Y_{1:M}$ with $f^{Y_{1:M}}(y_{1:M})>0$ be given. For each time *t*∈ [*t*_0_,*∞*) and for each *j*∈{1,…,*M*}, we define random variables

$$W_{j,t}:\Omega \to R_{\geq 0}\;\text{and}\;W_{t}:\Omega \to R_{\geq 0}$$

by the following system of ODEs

(28) $$\begin{array}{lll} \;dW_{1,t}(\omega ) & =\left(g_{1}(y_{1}\;|\;X_{t}(\omega ),t)-1\right)\gamma_{1}(t)\;\;dt\;\\ & \vdots \; & \;\\ \;dW_{M,t}(\omega ) & =\left(g_{M}(y_{M}\;|\;X_{t}(\omega ),t)-1\right)\gamma_{M}(t)\;\;dt\; & \; \end{array}$$

for each *ω*∈*Ω* with initial values

(29) $$W_{1,t_{0}}(\omega )=\cdots =W_{M,t_{0}}(\omega )=1,$$

and by

(30) $$W_{t}=\overset{M}{\underset{j=1}{\prod }}W_{j,t}.$$

We will show that for each set *A* in the *σ*-algebra generated by the variable *X*_*t*_, it holds that

(31) $$\;\begin{array}{c} \int_{X_{t}(A)}{\hat{f}}_{t}^{X_{t}\;|\;Y_{1:M}}(x_{t}\;|\;y_{1:M})\;\;dL_{X_{t}}(x_{t})=\frac{\int_{A}W_{t}(\omega )\;d\text{P}(\omega )}{\int_{\Omega }W_{t}(\omega )\;\;d\text{P}(\omega )} \end{array}$$

where ${\hat{f}}_{t}^{X_{t}\;|\;Y_{1:M}}$ is the filter density. That means we can use the processes *W*_*j*,*t*_ and *W*_*t*_ to compute the filter distributions through time. From this, it follows immediately that we can also compute filter expectations. Indeed, for any real-valued measurable function *h* on X such that E[|*h*(*X*_*t*_)|]<*∞*, it holds that the expectation of *h*(*X*_*t*_) given *Y*_1:*M*_=*y*_1:*M*_ with respect to the filtered state *X*_*t*_ defined by

(32) $$\begin{array}{c} {\hat{\text{E}}}_{t}[h(X_{t})\;|\;Y_{1:M}=y_{1:M}]:={\text{E}}_{{\hat{f}}_{t}^{X_{t}\;|\;Y_{1:M}}(\cdot \;|\;y_{1:M})}[h(X_{t})]\\ \;=\;\;\int_{X_{t}}{\hat{f}}_{t}^{X_{t}\;|\;Y_{1:M}}(x_{t}\;|\;y_{1:M})h(x_{t})\;\;dL_{X_{t}}(x_{t}), \end{array}$$

is given by the following equation:

(33) $$\begin{array}{c} {\hat{\text{E}}}_{t}\left[h(X_{t})\;|\;Y_{1:M}=y_{1:M}\right]=\frac{\int_{\Omega }W_{t}(\omega )h(X_{t}(\omega ))\;d\text{P}(\omega )}{\int_{\Omega }W_{t}(\omega )\;\;d\text{P}(\omega )}. \end{array}$$

To show our assertion, we consider the processes *W*_*j*,*t*_ for *j*=1,…,*M*. According to (28) and (29), each *W*_*j*,*t*_ is defined as

(34) $$W_{j,t}=1+\int_{t_{0}}^{t}\left(g_{j}(y_{j}\;|\;X_{t_{j}},t_{j})-1\right)\gamma_{j}(t_{j})\;\;dt_{j},$$

so, with (30),

(35) $$\begin{array}{c} W_{t}=\overset{M}{\underset{j=1}{\prod }}W_{j,t}=\overset{M}{\underset{j=1}{\prod }}\left(1+\int_{t_{0}}^{t}\left(g_{j}(y_{j}\;|\;X_{t_{j}},t_{j})-1\right)\gamma_{j}(t_{j})\;\;dt_{j}\right) \end{array}$$

holds. Thus, to prove (31), we have to show that for each set *A* from the *σ*-algebra generated by *X*_*t*_,

$$\begin{array}{lll} \frac{\int_{A}W_{t}(\omega )\;d\text{P}(\omega )}{\int_{\Omega }W_{t}(\omega )\;d\text{P}(\omega )} & =\left(\int_{X_{t}(A)}\int_{\left\{{\tilde{x}}_{\left[t_{0},t\right]}\in X_{\left[t_{0},t\right]}\;:\;{\tilde{x}}_{t}=x_{t}\right\}}\right)\; & \;\\ & \overset{M}{\underset{j=1}{\prod }}\;\left(1\;+\;\int_{t_{0}}^{t}\left(g_{j}(y_{j}\;|\;{\tilde{x}}_{t_{j}},t_{j})\;-\;1\right)\gamma_{j}(t_{j})\;\;dt_{j}\right)\; & \;\\ & \times dL_{X_{\left[t_{0},t\right]}}({\tilde{x}}_{\left[t_{0},t\right]})\;dL_{X_{t}}(x_{t}))\;\;\;/{\hat{f}}_{t}^{Y_{1:M}}(y_{1:M})\; & \; \end{array}$$

holds (see (25) and (26)). It is enough to show the equality for the numerator, i.e.

$$\begin{array}{c} \int_{A}W_{t}(\omega )\;d\text{P}(\omega )=\int_{X_{t}(A)}\int_{\left\{{\tilde{x}}_{\left[t_{0},t\right]}\in X_{\left[t_{0},t\right]}\;:\;{\tilde{x}}_{t}=x_{t}\right\}}\\ \;\overset{M}{\underset{j=1}{\prod }}\left(1+\int_{t_{0}}^{t}\left(g_{j}(y_{j}\;|\;{\tilde{x}}_{t_{j}},t_{j})-1\right)\gamma_{j}(t_{j})\;dt_{j}\right)\;\\ \;\times dL_{X_{\left[t_{0},t\right]}}({\tilde{x}}_{\left[t_{0},t\right]})\;dL_{X_{t}}(x_{t}) \end{array}$$

since the equality of the denominator follows then immediately from the special case *A*=*Ω* and from the fact that

$$\begin{array}{lll} \;{\hat{f}}_{t}^{Y_{1:M}}(y_{1:M})= & \;\int_{X_{t}}\int_{\left\{{\tilde{x}}_{\left[t_{0},t\right]}\in X_{\left[t_{0},t\right]}\;:\;{\tilde{x}}_{t}=x_{t}\right\}}\; & \;\\ & \overset{M}{\underset{j=1}{\prod }}\left(1+\int_{t_{0}}^{t}\left(g_{j}(y_{j}\;|\;{\tilde{x}}_{t_{j}},t_{j})-1\right)\gamma_{j}(t_{j})\;dt_{j}\right)\;\; & \;\\ & \times dL_{X_{\left[t_{0},t\right]}}\left({\tilde{x}}_{\left[t_{0},t\right]}\right)\;dL_{X_{t}}(x_{t}).\; & \; \end{array}$$

Using the variable transformation $\left(X_{\left[t_{0},t\right]}(\omega ),X_{t}(\omega ))=({\tilde{x}}_{\left[t_{0},t\right]},x_{t}\right)$, we get

$$\begin{array}{c} \int_{A}W_{t}(\omega )\;d\text{P}(\omega )\\ \;=\int_{A}\overset{M}{\underset{j=1}{\prod }}\left(1+\int_{t_{0}}^{t}\left(g_{j}(y_{j}\;|\;X_{t_{j}}(\omega ),t_{j})-1\right)\gamma_{j}(t_{j})\;dt_{j}\right)\;d\text{P}(\omega )\\ \;=\int_{X_{t}(A)}\int_{\left\{{\tilde{x}}_{\left[t_{0},t\right]}\in X_{\left[t_{0},t\right]}\;:\;{\tilde{x}}_{t}=x_{t}\right\}}\\ \;\;\overset{M}{\underset{j=1}{\prod }}\left(1+\int_{t_{0}}^{t}\left(g_{j}(y_{j}\;|\;{\tilde{x}}_{t_{j}},t_{j})-1\right)\gamma_{j}(t_{j})\;dt_{j}\right)\\ \;\times dL_{X_{\left[t_{0},t\right]}}({\tilde{x}}_{\left[t_{0},t\right]})\;dL_{X_{t}}(x_{t}). \end{array}$$

This is what we wanted to show.

#### Weights

Since for each *t* and for each *j*=1,…,*M* the random variables *W*_*j*,*t*_ and *W*_*t*_ depend only on the process $x_{\left[t_{0},t\right]}$ until time *t*, we can define functions $w_{j,t}:X_{\left[t_{0},t\right]}(\Omega )\to R_{\geq 0}$ and $w_{t}:X_{\left[t_{0},t\right]}(\Omega )\to R_{\geq 0}$ by setting for each $x_{\left[t_{0},t\right]}\in X_{\left[t_{0},t\right]}(\Omega )\subset X_{\left[t_{0},t\right]}$:

$$\begin{array}{c} w_{j,t}(x_{\left[t_{0},t\right]}):=W_{j,t}(\omega )\;\text{and}\;w_{t}(x_{\left[t_{0},t\right]}):=W_{t}(\omega )\\ \;\text{for some}\;\;\omega \in \Omega \;\;\text{with}\;\;X_{\left[t_{0},t\right]}(\omega )=x_{\left[t_{0},t\right]}. \end{array}$$

It follows from (34) that

(36) $$w_{j,t}(x_{\left[t_{0},t\right]})=1+\int_{t_{0}}^{t}\left(g_{j}(y_{j}\;|\;x_{t_{j}},t_{j})-1\right)\gamma_{j}(t_{j})\;dt_{j}$$

for each *j* and from (35) that

(37) $$w_{t}(x_{\left[t_{0},t\right]})=\overset{M}{\underset{j=1}{\prod }}w_{j,t}(x_{\left[t_{0},t\right]}).$$

The values of *W*_*t*_ will serve as weights in the MTU particle filter. We will call *W*_*j*,*t*_ the partial weights. Since in each discretization scheme which is applied to solve the integral in the formula (36) for *W*_*j*,*t*_ the integrand has to be evaluated, we may run into practical problems if we use it as it is written in the formula. If the density $g_{j}(y_{j}\;|\;x_{t_{j}},t_{j})$ evaluates to a very small value and if we work with fixed-precision numbers, the subtraction of 1 will result in a value which may be practically equal to -1. If this error accumulates over time, we may end up with wrong values for $w_{j,t}(x_{\left[t_{0},t\right]})$. To reduce the computational error, the integral could be split up in the following way:

(38) $$\begin{array}{ll} \;w_{j,t}(x_{\left[t_{0},t\right]})=1-\int_{t_{0}}^{t}\gamma_{j}(t_{j})\;dt_{j}+\int_{t_{0}}^{t}g_{j}(y_{j}\;|\;x_{t_{j}},t_{j})\gamma_{j}(t_{j})\;dt_{j}\; & \;\\ \;=1-{\bar{\gamma }}_{j,t}+{\bar{w}}_{j,t}(x_{\left[t_{0},t\right]})\; \end{array}$$

where the cumulative distribution function

(39) $${\bar{\gamma }}_{j,t}:=\int_{t_{0}}^{t}\gamma_{j}(t_{j})\;dt_{j}$$

is independent of $x_{\left[t_{0},t\right]}$ and where the part

(40) $${\bar{w}}_{j,t}(x_{\left[t_{0},t\right]}):=\int_{t_{0}}^{t}g_{j}(y_{j}\;|\;x_{t_{j}},t_{j})\gamma_{j}(t_{j})\;dt_{j}$$

depends on the path $x_{\left[t_{0},t\right]}$. It is even more convenient to compute ${\bar{\gamma }}_{j,t}$ by evaluating the antiderivative of *γ*_*j*_, if it is computationally available.

Note that the definition of the filter distribution is dependent on the reference measure $\mu_{Y_{j}}$. A suitable change of this measure may help to further increase the efficiency of the algorithm. This issue still has to be explored.

#### Resampling

Special attention is needed for the computation of the weights after resampling steps have been applied. As mentioned earlier, resampling at time *s*_*ℓ*_ is done by randomly generating a selection function *ι*_*ℓ*_:*I*→*I* (with index set *I*={1,…,*N*}) based on given non-negative (unnormalized) selection weights $v_{s_{\ell }}^{i}$ for each particle index *i*. The state samples ${\left(x_{s_{\ell }}^{i}\right)}_{i=1,\ldots ,N}$ have then to be replaced by the selected state samples ${\left(x_{s_{\ell }}^{\iota_{\ell }(i)}\right)}_{i=1,\ldots ,N}$, and the unnormalized weights ${\left(w_{s_{\ell }}^{i}\right)}_{i=1,\ldots ,N}$ by the corrected weights ${\left(w_{s_{\ell }}^{\iota_{\ell }(i)}/v_{s_{\ell }}^{\iota_{\ell }(i)}\right)}_{i=1,\ldots ,N}$. We assume that resampling steps at times *s*_1_,…,*s*_*ℓ*_ with *t*_0_≤*s*_1_⋯<*s*_*ℓ*_≤*t* have occurred, states and weights have been replaced at these times, and within this paragraph, we denote them by $x_{t}^{s_{1},\ldots ,s_{\ell };i}$ and $w_{t}^{s_{1},\ldots ,s_{\ell }}(x_{\left[t_{0}:t\right]}^{s_{1},\ldots ,s_{\ell };i})$, respectively, for each particle *i*. By definition, we have at *t*=*s*_*ℓ*_

$$x_{s_{\ell }}^{s_{1},\ldots ,s_{\ell };i}=x_{s_{\ell }}^{s_{1},\ldots ,s_{\ell -1};\iota_{\ell }(i)},$$

and

$$w_{s_{\ell }}^{s_{1},\ldots ,s_{\ell }}\left(x_{\left[t_{0}:s_{\ell }\right]}^{s_{1},\ldots ,s_{\ell };i}\right)=\frac{w_{s_{\ell }}^{s_{1},\ldots ,s_{\ell -1}}\left(x_{\left[t_{0}:s_{\ell }\right]}^{s_{1},\ldots ,s_{\ell };i}\right)}{v_{s_{\ell }}^{\iota_{\ell }(i)}}.$$

For each time *t*≥*s*_*ℓ*_ and for each particle *i*, the corrected weights are then recursively given by

(41) $$\begin{array}{c} w_{t}^{s_{1},\ldots ,s_{\ell }}\left(x_{\left[t_{0}:t\right]}^{s_{1},\ldots ,s_{\ell };i}\right)=\frac{w_{t}^{s_{1},\ldots ,s_{\ell -1}}\left(x_{\left[t_{0}:t\right]}^{s_{1},\ldots ,s_{\ell };i}\right)}{v_{s_{\ell }}^{\iota_{\ell }(i)}}=\frac{w_{t}\left(x_{\left[t_{0}:t\right]}^{s_{1},\ldots ,s_{\ell };i}\right)}{\prod_{\lambda =1}^{\ell }v_{s_{\lambda }}^{\iota_{\lambda :\ell }(i)}} \end{array}$$

with

$$\iota_{\lambda :\ell }(i)=\iota_{\lambda }\circ \cdots \circ \iota_{\ell }(i).$$

Since the process *W*_*t*_ computes the uncorrected weights $w_{t}(x_{\left[t_{0}:t\right]}^{s_{1},\ldots ,s_{\ell };i})$, we have to correct the weights explicitly in the algorithm by dividing them by the cumulative product

$${\bar{v}}_{t}^{s_{1},\ldots ,s_{\ell };i}:=\overset{\ell }{\underset{\lambda =1}{\prod }}v_{s_{\lambda }}^{\iota_{\lambda :\ell }(i)}.$$

Note that we have to select these products during a resampling step similarly to the selection of the states, i.e. at *t*=*s*_*ℓ*_,

$${\bar{v}}_{s_{\ell }}^{s_{1},\ldots ,s_{\ell };i}={\bar{v}}_{s_{\ell }}^{s_{1},\ldots ,s_{\ell -1};\iota_{\ell }(i)}v_{s_{\ell }}^{\iota_{\ell }(i)}.$$

#### The MTU particle filter algorithm

A practical MTU particle filter can be obtained by any discretization scheme based on an (arbitrary) time discretization *t*_0_ = *τ*_0_*τ*_1_…*τ*_*D*_. Similar to the standard particle filter, sampling need not necessarily be done from the state process $X_{\left[t_{0},\infty \right)}$ directly. One can instead sample from another process ${\tilde{X}}_{\left[t_{0},\infty \right)}$, provided that the Radon-Nikodym derivatives

$$\begin{array}{c} \varrho_{t_{0}}(x_{t_{0}}):=\frac{dL_{X_{t_{0}}}(x_{t_{0}})}{dL_{{\tilde{X}}_{t_{0}}}(x_{t_{0}})}\;\text{and}\;\varrho_{t\;|\;s}(x_{t}\;|\;x_{s}):=\frac{K_{s,t}(x_{s},\;dx_{t})}{{\tilde{K}}_{s,t}(x_{s},\;dx_{t})} \end{array}$$

for each *s*,*t*∈[*t*_0_,*t*] with *s*<*t* exist and can be evaluated pointwise. (In fact, it suffices that *ϱ*_*t* | *s*_(*x*_*t*_ | *x*_*s*_) exists for all states $x_{s}\in X_{s}$ which are reachable from some initial state $x_{t_{0}}\in X_{t_{0}}$ with $\varrho_{t_{0}}(x_{t_{0}})\neq 0$). In the special case that we sample from the Markov kernels of $X_{\left[t_{0},\infty \right)}$ directly, *ϱ*_*t* | *s*_≡1 for all *s*<*t*. Our MTU particle filter is described in algorithm 2. Here we suppress the indices *s*_1_,…,*s*_*ℓ*_ in the notations of states and weights (see last paragraph).

##### Algorithm 2 MTU particle filter

The algorithm as it is written here has to be enriched with concrete discretization methods for the sampling and update steps. For instance, if the process ${\tilde{X}}_{\left[t_{0},\infty \right)}$ is a multidimensional Itô process defined through a stochastic differential equation (SDE)

$$d{\tilde{X}}_{t}=a({\tilde{X}}_{t},t)\;dt+B({\tilde{X}}_{t},t)\;dW_{t}$$

with drift *a*(*x*,*t*), diffusion matrix *B*(*x*,*t*), multidimensional standard Wiener process $W_{t}$, and initial variable ${\tilde{X}}_{t_{0}}$, then the Euler-Maruyama method can be used for discretization. We set *Δτ*_*d*_: =*τ*_*d*_-*τ*_*d*-1_. The sampling is then done by

(42) $$x_{\tau_{d}}^{i}=a\left(x_{\tau_{d-1}}^{i},\tau_{d-1}\right)\Delta \tau_{d}+B\left(x_{\tau_{d-1}}^{i},\tau_{d-1}\right)\sqrt{\Delta \tau_{d}}\;\eta^{i}$$

where *η*^*i*^ is a sample from a standard normal distribution (with mean 0 and variance 1). Further, the update step can be done in the simplest case using Euler discretization:

(43) $${\bar{\gamma }}_{j,\tau_{d}}={\bar{\gamma }}_{j,\tau_{d-1}}+\gamma_{j}(\tau_{d-1})\Delta \tau_{d}$$

and

(44) $${\bar{w}}_{j,\tau_{d}}^{i}={\bar{w}}_{j,\tau_{d-1}}^{i}+g_{j}(y_{j}\;|\;x_{\tau_{d-1}}^{i},\tau_{d-1})\gamma_{j}(\tau_{d-1})\Delta \tau_{d}.$$

Of course, better discretization schemes are possible. As we have already mentioned, if the antiderivative *G*_*j*_ with *G*_*j*_(*t*_0_)=0 (which is in fact the distribution function) of *γ*_*j*_ is available, then one should rather use

$${\bar{\gamma }}_{j,\tau_{d}}=G_{j}(\tau_{d})$$

for the computation of the values ${\bar{\gamma }}_{j,\tau_{d}}$.

#### Adaptive stepsize

To be able to fully exploit our MTU particle filter method, the discretization stepsize must be chosen appropriately. One simple possibility is to use a very small stepsize throughout the complete procedure. A quite high computation time will result from that. This can be reduced if an adaptive stepsize is chosen. We propose to determine the stepsize *Δτ*_*d*_ online depending on the ESS estimate. The stepsize should decrease when the ESS drops rapidly, and it should increase again if the ESS estimate changes only marginally. In detail, the following procedure can be applied.

In each step of the algorithm, we obtain an initial guess of the stepsize by a linear interpolation between a maximal stepsize if the ESS had not changed since the last step, and a minimal stepsize if the ESS had dropped by the number *N* of samples (actually, the maximal difference that can be obtained is *N*-1). From this initial guess, we compute the increments of the partial weights, and from them we predict the ESS in the next step based on the current stepsize guess. If the difference between this predicted ESS and the current ESS drops by more than a certain relative amount (we use 10%), then a new guess of the stepsize is computed by dividing the current guess by 2. With this new guess, a new predicted ESS is computed, and the test can be applied again. This procedure will be applied iteratively until either the difference between predicted and current ESS drops by less than the prescribed amount, or the stepsize guess falls below a prescribed minimal stepsize. The current stepsize or the minimal stepsize, respectively, is then accepted, and the algorithm proceeds with this stepsize. See algorithm 3 for a formal description of the stepsize determination.

##### Algorithm 3 Determination of the adaptive stepsize

If we sample from the Markov kernels of $X_{\left[t_{0},\infty \right)}$ directly, then $\varrho_{\tau_{d}^{*}\;|\;\tau_{d-1}}\left(x_{\tau_{d}^{*}}^{i}\;|\;x_{\tau_{d-1}}^{i}\right)\equiv 1$ and the update of the weights does not depend on the new states $x_{\tau_{d}^{*}}^{i}$. Hence we only need to compute the weight update and the corresponding ESS estimate until we find an adequate stepsize. In this case, it is not necessary to sample the new states in each iteration which renders the algorithm computationally more effective.

Note that this procedure cannot be performed when the measurement times are fixed (i.e. in the standard particle filter). In this case the ESS does not depend on the stepsize, and a reduction of it will not improve the ESS. The application of the MTU particle filter with distributed measurement times is essential to be able to use this adaptive stepsize procedure.

#### Data Likelihood

As mentioned earlier for the standard case of the particle filter algorithm, without resampling, the data likelihood could be approximated by the empirical mean of the unnormalized weights (see (19)). In our case, this would be

$${\hat{Z}}_{t}:=\frac{1}{N}\overset{N}{\underset{i=1}{\sum }}w_{t}^{i}=\frac{1}{N}\overset{N}{\underset{i=1}{\sum }}w_{t}(x_{t}^{i}).$$

After each resampling step, we have to correct this formula. Using the same notation as in the paragraph on resampling, for each time *t*≥*s*_*ℓ*_ and for each particle *i*, the corrected estimate is given by

(45) $$\begin{array}{c} {\hat{Z}}_{t}:=\frac{1}{N^{\ell +1}}\left(\overset{\ell }{\underset{\lambda =1}{\prod }}\overset{N}{\underset{i=1}{\sum }}v_{s_{\lambda }}^{i}\right)\overset{N}{\underset{i=1}{\sum }}w_{t}^{s_{1},\ldots ,s_{\ell }}\left(x_{\left[t_{0}:t\right]}^{s_{1},\ldots ,s_{\ell };i}\right)\\ \;=\frac{1}{N^{\ell +1}}\left(\overset{\ell }{\underset{\lambda =1}{\prod }}\overset{N}{\underset{i=1}{\sum }}v_{s_{\lambda }}^{i}\right)\overset{N}{\underset{i=1}{\sum }}\frac{w_{t}\left(x_{\left[t_{0}:t\right]}^{s_{1},\ldots ,s_{\ell };i}\right)}{\overset{\ell }{\underset{\lambda =1}{\prod }}v_{s_{\lambda }}^{\iota_{\lambda :\ell }(i)}} \end{array}$$

with

$$\iota_{\lambda :\ell }(i)=\iota_{\lambda }\circ \cdots \circ \iota_{\ell }(i)$$

(see (41)). This can be seen by considering recursively the correction at the time of the resampling step *ℓ*. Since

$$\frac{Nv_{s_{\ell }}^{\iota_{\ell }(i)}}{\sum_{\nu =1}^{N}v_{s_{\ell }}^{\nu }}$$

is the expected number of times the particle *i* has been selected after *N* draws, we have to correct the weights $w_{s_{\ell }}^{s_{1},\ldots ,s_{\ell -1}}\left(x_{\left[t_{0}:s_{\ell }\right]}^{s_{1},\ldots ,s_{\ell };i}\right)$ in selection step *ℓ* by dividing them by this number. The following computation then proves the correctness of the formula via induction:

$$\begin{array}{c} {\hat{Z}}_{s_{\ell }}=\frac{1}{N^{\ell }}\left(\overset{\ell -1}{\underset{\lambda =1}{\prod }}\overset{N}{\underset{i=1}{\sum }}v_{s_{\lambda }}^{i}\right)\\ \;\times \overset{N}{\underset{i=1}{\sum }}\left(w_{s_{\ell }}^{s_{1},\ldots ,s_{\ell -1}}\left(x_{\left[t_{0}:s_{\ell }\right]}^{s_{1},\ldots ,s_{\ell };i}\right)/\frac{Nv_{s_{\ell }}^{\iota_{\ell }(i)}}{\overset{N}{\underset{\nu =1}{\sum }}v_{s_{\ell }}^{\nu }}\right)\\ \;=\frac{1}{N^{\ell +1}}\left(\overset{\ell }{\underset{\lambda =1}{\prod }}\overset{N}{\underset{i=1}{\sum }}v_{s_{\lambda }}^{i}\right)\overset{N}{\underset{i=1}{\sum }}\frac{w_{s_{\ell }}^{s_{1},\ldots ,s_{\ell -1}}\left(x_{\left[t_{0}:s_{\ell }\right]}^{s_{1},\ldots ,s_{\ell };i}\right)}{v_{s_{\ell }}^{\iota_{\ell }(i)}}\\ \;=\frac{1}{N^{\ell +1}}\left(\overset{\ell }{\underset{\lambda =1}{\prod }}\overset{N}{\underset{i=1}{\sum }}v_{s_{\lambda }}^{i}\right)\overset{N}{\underset{i=1}{\sum }}w_{s_{\ell }}^{s_{1},\ldots ,s_{\ell }}\left(x_{\left[t_{0}:s_{\ell }\right]}^{s_{1},\ldots ,s_{\ell };i}\right). \end{array}$$

We get algorithm 4 for the computation of the empirical estimate of the data likelihood, which needs to be done in parallel to the MTU particle filter algorithm.

##### Algorithm 4 Estimation of the data likelihood

#### Offline and online estimation

Two main cases of estimation procedures may be distinguished: the offline estimation procedure used for example for parameter estimation, and the online estimation procedure for control purposes. While in the offline case the measurements are completely available before estimation begins, we know only some of the measurement values at some certain time *t* during the online procedure. In this latter case, an online computation where all measurement times have been modelled with probability densities with infinite support is impossible, because in this situation all measurement values must already be known at time *t*_0_. Online estimation is nevertheless possible if the support of the measurement time densities is finite. The online estimation must then be delayed by the diameter of the respective supports.

#### Implementation

We have implemented the proposed algorithm in Mathematica as part of a Parameter Estimation Toolbox for Systems Biology developed by the Systems Biology group at the Fraunhofer Chalmers Centre (FCC) in Göteborg (Sweden). Furthermore, we have implemented it also in the statistical computing language R [28]. All figures in this article have been created using this R implementation.

## Results and Discussion

### Motivating example - results

**Figure 2:**
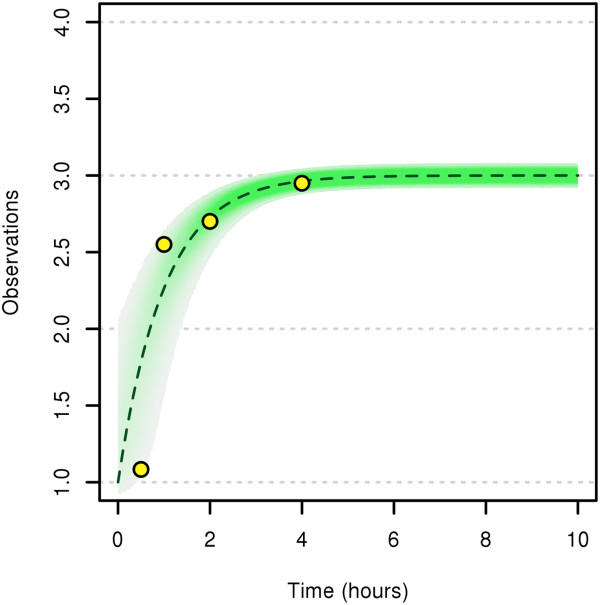
**Measurement distribution with a set of possible measurements.** The dashed dark-green line depicts the nominal evolution of the state *q* over time. The green shaded area depicts the region where the measurements are expected. The yellow circles depict a set of possible measurement values.

In this section, we resume our motivating example and use it in a parameter estimation setting to compare our MTU filter to both the standard particle filter and to a state-of-the-art Maximum Likelihood (ML) method which is not based on Monte Carlo techniques. To this aim, we first create virtual “measurements” at four intended measurement times ${\hat{t}}_{j}$, *j*=1,…,4 by simulation runs with our true model (see Table 1). This is done as follows: We simulate a single state path (*q*(*t*))_*t*∈[0,10]_ based on the “true” parameter values *α*=1 and *β*=3. Then, for each intended measurement time ${\hat{t}}_{j}$, we sample an actual measurement time *T*_*j*_ according to the density *γ*_*j*_. Now, for each *T*_*j*_, we sample a measurement value *y*_*j*_ from the distribution $N(q(t_{j}),\sigma_{y}^{2})$. Figure 2 shows one set of measurement values obtained in this way. The intended measurement times are 0.5, 1, 2, 4 and the measurement values we got from one simulation run are 1.083346, 2.550290, 2.700863, 2.949450. We will use these values in the following estimation runs. Our aim is to estimate the parameter vector *θ*=(*α*,*β*) with the following three differrent estimation procedures and compare the results: 

1. ML estimation on “lumped” measurements,

2. Standard PF on “lumped” measurements, and

3. MTU-PF.

Note that in both the standard PF and the ML estimation method, it is not possible to include the uncertainties in the measurement times directly. Rather, we have to lump these uncertainties with the uncertainties in the measurement values by increasing their variances $\sigma_{y}^{2}$ and to treat the ${\hat{t}}_{j}$ like true measurement times. In both cases, we tested several values of “lumped” variances $\sigma_{y}^{2}$.

We have implemented the standard particle filter and the MTU particle filter in the statistical computing language R [28]. The ML estimations have been done using the Parameter Estimation Toolbox for Systems Biology developed by the Systems Biology group at the Fraunhofer Chalmers Centre (FCC) in Göteborg (Sweden).

#### ML estimation

The ML estimation is a standard method based on a Maximum Likelihood approach, that is, on the maximization of the likelihood function *L*(*θ*;*y*_1:*M*_) with respect to the parameter vector *θ*. The computation of the likelihood requires the use of estimates of the state mean and covariance matrix which are routinely obtained by the use of a non-linear derivation of the Kalman filter.

Given measurements *Y*_1:*M*_ at a total of *M* time points *T*_*j*_ which are assumed to be fixed and known, the likelihood function can be written as

(46) $$\begin{array}{c} L(\theta ;y_{1:M}):=f^{Y_{1:M},T_{1:M},\theta }(y_{1:M};\theta )\\ \;=\overset{M}{\underset{j=1}{\prod }}f^{Y_{j}|Y_{1:j-1},T_{1:M},\theta }(y_{j}|y_{1:j-1},t_{1:M};\theta ) \end{array}$$

where $f^{Y_{j}|Y_{1:j-1},T_{1:M},\theta }$ is the conditional probability density function of the *j*-th observation *y*_*j*_ given all previous observations *y*_1:*j*-1_ as given in (9), here explicitly based on the given parameter vector *θ*. As already mentioned, analytical solutions are not generally available. An exception to this is the case that all measurements are conditionally Gaussian, that means that all conditional densities $f^{Y_{j}|Y_{1:j-1},T_{1:M},\theta }$ are Gaussian. In this case, the Kalman filter yields the correct solution. The assumption that the measurements are conditionally Gaussian is commonly used as an approximation even in those cases where this is not true. If one accepts that this assumption gives an approximation which is close enough to the true model, then it is only necessary to propagate the means and covariances of the measurements, since the Gaussian probability distribution is completely characterized by the first two moments. We introduce the notation

$${ŷ}_{j|j-1}=\text{E}[y_{j}|y_{1:j-1};\theta ]$$

and

$$R_{j|j-1}=\mathrm{Cov}[y_{j}|y_{1:j-1};\theta ]$$

for the prediction of the mean and covariance of the observation variables, respectively. The computation of these values can be achieved by some derivates of the Kalman filter, commonly used are the Extended Kalman Filter (EKF) or the Unscented Kalman Filter (UKF), more exactly those versions of these filters which are time-continuous in the states and time-discrete in the measurements (continuous/discrete EKF and UKF, respectively). The residuals *ϵ*_*j*_ are then defined by the differences between the measurements *y*_*j*_ and their estimations ${ŷ}_{j|j-1}$:

$$\epsilon_{j}:=y_{j}-{ŷ}_{j|j-1}.$$

The assumption of normally distributed observations leads to an approximation of the likelihood function of the following form

$$L(\theta ;y_{1:M})\approx \hat{L}(\theta ;y_{1:M}):=\overset{M}{\underset{j=1}{\prod }}\frac{\exp\left(-\frac{1}{2}\overset{T}{\underset{j}{\epsilon }}R_{j|j-1}^{-1}\epsilon_{j}\right)}{\sqrt{\left|R_{j|j-1}\right|}{\left(\sqrt{2\Pi }\right)}^{l}}$$

where *l* is the number (dimension) of the observation variables. The negative logarithm of this approximated likelihood function is

$$\begin{array}{ll} -\log\hat{L}(\theta ;y_{1:M})=\frac{1}{2}\overset{M}{\underset{j=1}{\sum }}\left(\log(\det(R_{j|j-1}))+\overset{T}{\underset{j}{\epsilon }}\overset{-1}{\underset{j|j-1}{R}}\epsilon_{j}\right)\; & \;\\ \;+\frac{\text{Ml}}{2}\log(2\Pi ).\; \end{array}$$

The problem to finding maximum likelihood estimates of the model parameters takes the form of a nonlinear optimization problem:

$$\hat{\theta }=\underset{\theta \in \Theta }{arg min}\{-\log\hat{L}(\theta ;y_{1:M})\}.$$

Roughly, the estimation with this approach is done as follows: 

1. Choose an initial guess *θ*^∗^ for the parameter vector *θ*.

2. Based on the current parameter vector *θ*^∗^ and for each (intended) measurement time *T*_*j*_, compute residuals *ϵ*_*j*_ and estimates of the covariance matrices *R*_*j*|*j*-1_ using (an approximation to) the Kalman filter.

3. Compute the likelihood *L* based on the state estimates *ϵ*_*j*_ and *R*_*j*|*j*-1_.

4. Use one step of some local optimization technique to find an improved parameter vector $\theta_{\text{new}}^{*}$ that decreases the negative log-likelihood function; replace *θ*^∗^ by this improved parameter vector $\theta_{\text{new}}^{*}$.

5. Repeat steps 2 to 4 until the parameter vector shows convergence.

**Figure 3:**
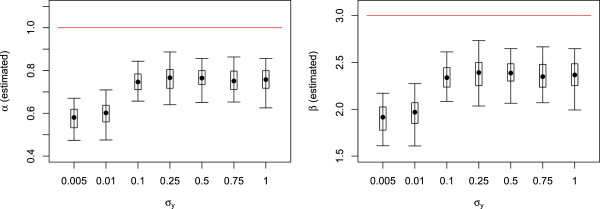
**Parameters*α* and*β* estimated by ML estimator.** Box plots of estimated parameters of 100 runs each. The medians are depicted with circles. The bottom and top of the box are the 0.25-quantile and the 0.75-quantile, i.e. 50% of the values lie within the box. The whiskers mark the 0.025-quantile and the 0.975-quantile, i.e. 95% of the values lie between the whiskers. The horizontal red lines denote the true parameter values.

The results of several runs of parameter estimations for different values of the lumped measurement variances $\sigma_{y}^{2}$ are shown in Figure 3. For each $\sigma_{y}^{2}$, we performed 100 runs with different initial state and parameter values. Note that the choice of an initial parameter value is mandatory for all approaches based on a local optimization method, and that different choices may lead to different results. On the other hand, in our example, we assume a log-normal prior distribution for the initial states (see Table 1), while the Kalman filter based approximations always assume normally distributed initial state values. Conditioned on the intial state (and current parameter), our example system is Gaussian linear which means that the Kalman filter yields exact estimates. We therefore decided the use the following procedure: Before each run, we sample the starting values for the parameters from the same priors as are available to the particle filter (see Table 2), and we also sample initial values *q*_0_ for the states from the correct distributions (see Table 1). Then we start the estimation procedure based on these initial parameter and state samples, and we keep the resulting estimated parameters as one “sample”. We repeat this procedure for all of the 100 runs. In this way, the standard procedure uses exact the same information as the particle filters, with the only exception of the measurement time distributions *γ*_*j*_. Figure 3 shows the distributions of the estimated parameter values as box plots.

**Table 2:** Choices for the PFs for the motivating example

	
prior for estimation of *α*	log-$N(\text{log}(2),1^{2})$
prior for estimation of *β*	log-$N(\text{log}(6),1^{2})$
stepsize for standard particle filter	10^-2^
maximal stepsize for MTU particle filter	10^-2^
minimal stepsize for MTU particle filter	10^-6^
diffusion parameter for artificial parameter noise at time *t*	5.43/(*t*+3.29)^2^

The estimated parameters of all estimation runs are far away from the true values; even quite large variances $\sigma_{y}^{2}$ do not improve the performance of the estimations. (We also tested the ML approach on a different data set of 4 measurements which was created *without* randomness in the measurement times: the ML approach performed much better in this case since the estimated parameters varied around the true values; data not shown).

#### Estimation with particle filters

Tests with the particle filters (both standard and MTU) have been performed in the following way: First we perform an *estimation run* where we use these measurements to estimate the parameters *α* and *β* of the system equation $dq(t)=(-\mathrm{\alpha q}(t)+\beta )\;dt+\sigma \;d\;W_{t}$. We perform this estimation with the MTU particle filter and with the standard particle filter under the same conditions and with the same seed for the random number generator. Thus the initial distribution of the particles is the same in both cases. For the standard filter, we use different levels of variance $\sigma_{y}^{2}$ for the measurement noise. Afterwards, the empirical medians of the final parameter distributions are used to perform a *simulation run* in each case, where we estimate the state *q* of the system based on the measurements and these fixed parameters. For both runs, we compute an estimate of the data likelihood over time. The choices of stepsizes and artificial parameter dynamics can be found in Table 2 (the values for the artificial parameter dynamics are computed by interpolation in the same way as we will describe later in the application of the MTU-PF to the plasma-leucine model).

Table 3 shows a comparison of the estimated parameters and data log-likelihood values. The estimated parameters (empirical medians) of the MTU particle filter (*α*=1.012 and *β*=3.010) are very close to the true values (*α*=1 and *β*=3) whereas the estimated values of the standard particle filter are significantly worse. The data log-likelihood in both estimation and simulation run is considerably higher for the MTU filter, compared to any of the standard filter runs.

**Table 3:** **Estimated parameters and data log-likelihood values for the MTU particle filter and the standard particle filter with different standard deviations*σ***_***y***_** for the measurement noise**

				**Data log-likelihood**	**Data log-likelihood**
	***σ***_***y***_	***α***	***β***	**Estimation run**	**Simulation run**
MTU	0.005	1.012	3.010	-4.327	2.398
standard	0.005	7.031	20.712	-24.084	-744.970
standard	0.01	9.102	26.919	-23.936	-890.746
standard	0.1	4.709	13.847	-9.081	-140.117
standard	0.25	1.425	4.171	-6.714	-4.618
standard	0.5	1.156	3.287	-5.538	-2.170
standard	0.75	1.318	3.604	-5.807	-3.160
standard	1	1.450	3.733	-6.227	-4.100

The true parameters are *α*=1 and *β*=3.

**Figure 4:**
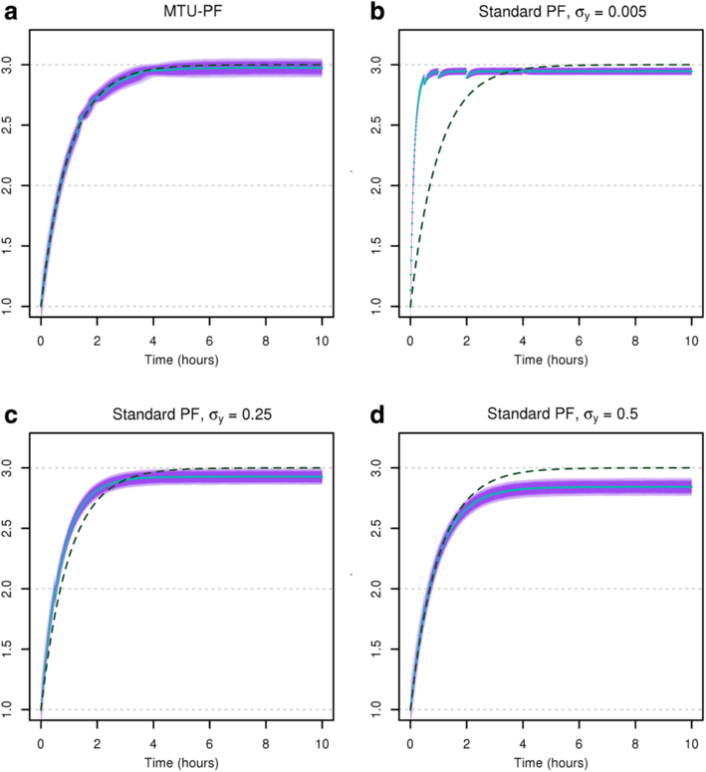
**Filtered state distributions based on simulation runs with estimated parameters for the motivating example.** MTU particle filter (**a**) and standard particle filter with several different assumed lumped measurement variances *σ*_*y*_ (**b**-**d**). Violet shaded area: Filtered state distributions based on empirical quantiles. Solid blue line: Median of filtered states. Dashed dark-green line: nominal evolution of the state *q* over time.

In Figure 4, we show the distributions of the filtered states obtained by simulation runs with the estimated parameters. The violet shaded area in Figure 4(a) depicts the estimated state distributions of the state *q* for the MTU particle filter with the light-blue line marking its median. The dashed dark-green line depicts the nominal evolution of the state *q* over time which should be approximated by the filter. Figures 4(b-d) show the estimated state distributions of the state *q* for the standard particle filter with different assumed lumped measurement variances. As can be seen from these figures, the approximation by the MTU particle filter is very close to the evolution of the state with the true parameters. On the contrary, however we choose the measurement variance in the standard case, the algorithm is not able to adequately approximate the correct state evolution.

**Figure 5:**
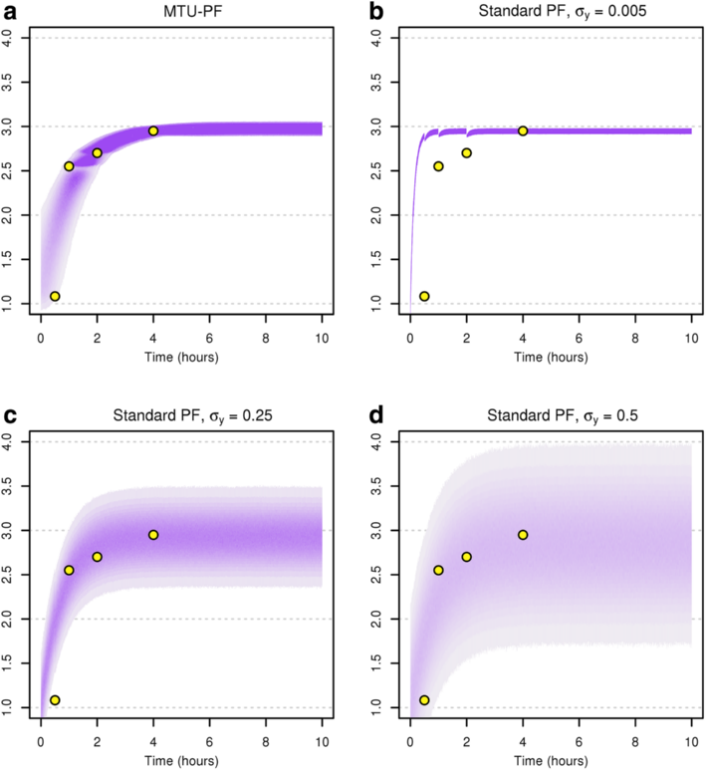
**Simulated measurements corresponding to the filtered state distributions for the motivating example.** MTU particle filter (**a**) and standard particle filter with several different assumed lumped measurement variances $\sigma_{y}^{2}$ (**b**-**d**). Violet shaded area: Measurement distributions based on empirical quantiles. Solid blue line: Median of simulated measurements. Yellow circles: Measurements.

Figure 5 displays the simulated measurement distributions corresponding to the filtered state distributions based on the simulation runs with estimated parameters. Here we notice again that the MTU particle filter can adapt well to the situation, whereas the measurement distributions which can be realized by the standard filter do not fit well with the data points.

**Figure 6:**
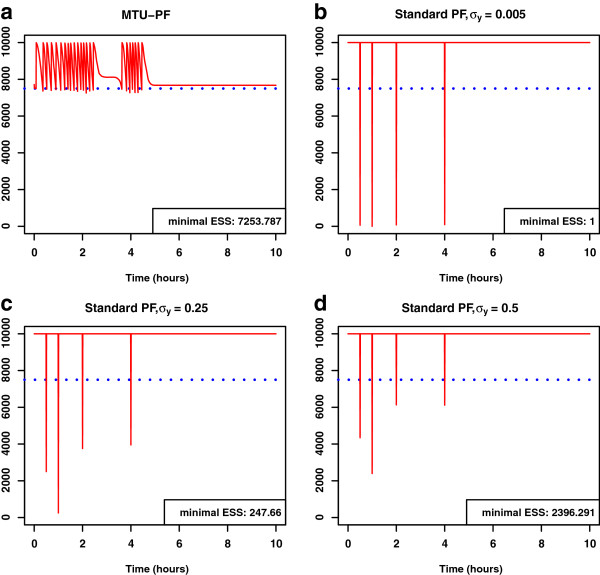
**Estimated effectice sample size during estimation run.** MTU particle filter (**a**) and standard particle filter with several different assumed lumped measurement variances *σ*_*y*_ (**b**-**d**). The dotted blue line denotes the resampling threshold.

In addition to the significant improvement in the parameter and state estimation, the MTU particle filter has another benefit. Figure 6 shows the development of the effective sample size estimate during the estimation runs. In the standard particle filter runs where the lumped measurement variance is assumed to be small, the predicted values for states and parameters do not fit well with the measurements. At the measurement times, when the predicted states are compared with the measurements and weighted accordingly, most weights decrease rapidly. This leads to a high variance of the weights and the effective sample size estimate drops severely, indicating that only very few particles effectively contribute to the estimation. This degeneration of the particle cloud is prevented by the MTU particle filter, as can be seen in Figure 6(a). For the standard filter runs where $\sigma_{y}^{2}$ is assumed to be larger, the ESS estimate does not drop as severely as with a small $\sigma_{y}^{2}$. This is due to the fact that, with a measurement variance which is assumed to be very high, more or less all possible simulated measurements are considered to fit well with the true measurements. However, these filters are not able to establish reasonably good estimates of the states and parameters and are clearly outperformed by the MTU particle filter.

### Application to the Plasma-Leucine Model with Population Data

In this section, we apply our MTU particle filter algorithm to a leucine kinetics model (Demant et al. [29] based on Cobelli et al. [30]) with data taken from a clinical study on diabetes patients ([31,32], see Additional file 1). We perform Bayesian population-based (i.e. NLME) parameter estimation with this model. The model and the data have been previously used in a maximum likelihood estimation context in [17]. It should be noted that the original ODE model needs to be extended by some kind of stochastic process variability in order to turn it into an SDE model. The approach taken in [17] is different from our approach in the way that in [17] the stochastic fluctuations are assumed to be in the tracee (plasma leucine) while we here assume the variability to be in the tracer (labelled leucine). Both assumptions are plausible; a final decision on the best way to model the process dynamics has not yet been made.

#### The Leucine model

**Figure 7:**
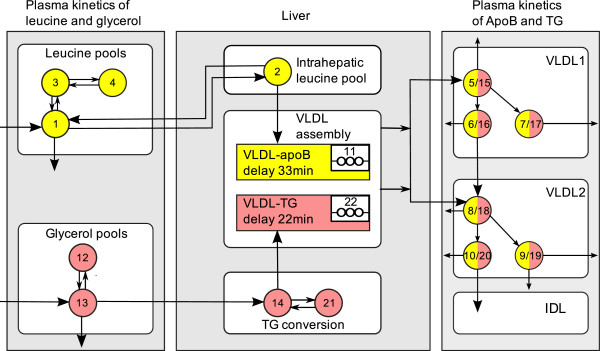
**Multicompartmental model for apolipoprotein B-100 (apoB) and triglyceride (TG) metabolism in very low density lipoprotein (VLDL) subfractions.** This multicompartmental model was developed in [31]. Circles depict compartments and arrows depict fluxes between compartments. The model includes separate modules for leucine (yellow) and glycerol (red). The free leucine plasma kinetics is modeled by two pools (3 and 4) and a plasma compartment (1), which interchange materials with an intrahepatic compartment (2). Compartment 2 feeds the apoB synthetic machinery. For glycerol, the plasma compartment (13) is connected to a pooling compartment (12) and feeds TG synthesis, which consists of a fast pathway (14) and a slow pathway (21). The assembly of lipoprotein is modeled by separate delays for apoB (11) and TG (22). The plasma kinetics of apoB and TG is modeled by a four-compartment hydrolysis chain, consisting of compartments 5, 6, 8, and 10 for apoB and compartments 15, 16, 18, and 20 for TG. Compartments 5/15 and 6/16 are associated with VLDL1, together with a slowly decaying compartment 7/17. Compartments 8/18 and 10/20 together with the slowly decaying compartment 9/19 form the VLDL2 module. Lipolysis of TG is modeled to take place in the transfer between two compartments. For details on the full model, see [31,34], or [33]. For our purposes, we use a restricted model consisting of the leucine pool, e.g. compartments 1 to 4, see also Figure 8.

In [31] (see also the thesis [33]), a new combined multicompartmental model for apolipoprotein B-100 (apoB) and triglyceride metabolism in very low density lipoprotein (VLDL) subfractions has been developed, see Figure 7. VLDL are transporters of triglycerides and cholesterol from the liver to the periphery, and elevated levels are associated with increased risk for cardiovascular events. Each VLDL particle has exactly one apoB molecule attached which makes apoB a suitable marker for triglyceride transport.

**Figure 8:**
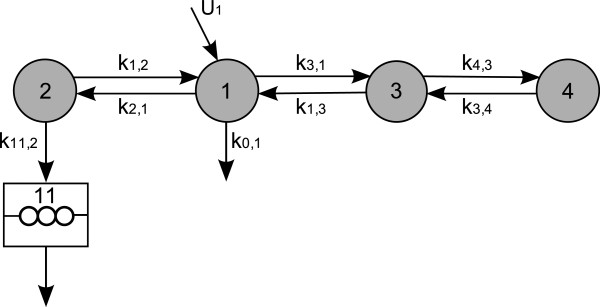
**Schematic depiction of the restricted model (leucine pool) [**[33]**].** This scheme is a subscheme of Figure 7. Circles depict compartments. Arrows depict fluxes between compartments and are labelled with the corresponding fractional transfer coefficients. Compartment 1 is the plasma-leucine compartment where the leucine is injected. Compartment 2 is an intrahepatic compartment which is the source for apoB synthesis. Compartments 3 and 4 are body protein pools. The output is at compartment 1. Compartment 11 is a delay compartment used only as output from compartment 2.

The secreted particles become denser and denser as triglycerides are delivered to target organs such as muscles and adipose tissue and the relative protein content is increased. As the density increases, the VLDL becomes an intermediate density lipoprotein (IDL) and finally a low density lipoprotein (LDL).

For our purposes we use only the part of the model concerning the leucine pool (compartments 1-4), see Figure 8. This subsystem was first used for apoB kinetic studies by Demant et al. [29] as an implementation of the work by Cobelli et al. [30]. The output is at compartment 1 and at compartment 2. The influx into compartment 1 will be denoted *U*_1_.

The data are obtained from tracer/tracee experiments. Here the tracee (i.e. the concentration we are actually interested in) is the leucine amino acids in the apoB molecules. Additional labelled leucine (tracer) is injected as a bolus infusion. Knowledge about the kinetics (fluxes between compartments) of the tracee can be gained by studying the kinetics of the tracer. In the restricted model four compartments are considered: plasma leucine (1), intra-hepatic leucine (2), and two plasma protein pools (3 and 4). In the full model, additional compartments represent VLDL subfractions (compartments 5-11 in Figure 7). The four-compartment system is linked to compartments 5-11 through compartment 2.

For each compartment *i* where *i*=1,…,4, let *Q*_*i*_ and *Q*_*i*_ denote the mass of the tracee and the tracer, respectively. Similarly, let *U*_*i*_ and *U*_*i*_ denote the input for the tracee and the tracer, respectively. For tracer/tracee experiments, *Q*_*i*_ is assumed to be in steady state. If the concentration level of the labelled injection is small compared with the overall concentration levels, and if the model is linear, then approximately

$$\frac{dq(t)}{dt}=K(t)q(t)+u(t)$$

where $Q(t)={\left(Q_{i}(t)\right)}_{i=1,2,3,4}^{T}$, *u*(*t*)=(*u*_1_(*t*),0,0,0)^T^ and

$$K(t)={\left(k_{j,i}\right)}_{j,i=1,2,3,4}$$

where *k*_*j*,*i*_ for *i*≠*j* is the transfer coefficient of the tracers from compartment *i* to compartment *j* (compartments 0 and 11 are considered to be output compartments), and for each *i*=1,…,4

$$k_{i,i}:=-\underset{\begin{array}{c} j=0,1,2,3,4,11\\ j\neq i \end{array}}{\sum }k_{j,i}.$$

Throughout this paper, the time unit used is hours, all fractional transfer coefficients are given in the unit h^-1^, and the amount of material in compartments is given in mg. In our model, only *k*_0,1_, *k*_1,2_, *k*_1,3_, *k*_2,1_, *k*_3,1_, *k*_3,4_, *k*_4,3_ and *k*_11,2_ are assumed to be non-zero, while additionally the following dependencies of the transfer coefficients are assumed to be valid:

$$\begin{array}{lll} k_{1,2} & =k_{2,1}\; & \;\\ k_{3,4} & =0.1\cdot k_{4,3}\; & \; \end{array}$$

The fractional transfer coefficient *k*_11,2_ has to be fixed for the system to be identifiable. We set *k*_11,2_=0.01h^-1^, as an estimated average from current results. We build stochastic differential equations (SDEs) from the resulting ordinary differential equations by adding noise terms which are given by standard Wiener processes $W_{1,t},\ldots ,W_{4,t}$ multiplied by diffusion parameters *σ*_1_,…,*σ*_4_, respectively. The leucine pool subsystem which we consider here (compartments 1-4) interacts with the surroundings only via an initial input into compartment 1 at time *t*=0, an output from compartment 1, and another output from compartment 2 (towards compartment 11). All other flows are processes acting inside the subsystem and hence should follow the principle of mass conservation. Therefore we add the stochastic terms to the ODE system in the following way:

$$\begin{array}{c} dq_{1}(t)=\;-(k_{1,2}+k_{0,1}+k_{3,1})\;(q_{1}(t)\;dt+\sigma_{1}\;dW_{1}(t))\\ \;+k_{1,2}\;(q_{2}(t)\;dt+\sigma_{2}\;dW_{2}(t))\\ \;+k_{1,3}\;(q_{3}(t)\;dt+\sigma_{3}\;dW_{3}(t)),\\ \;dq_{2}(t)=\;\;\;\;-(k_{11,2}+k_{1,2})\;(q_{2}(t)\;dt+\sigma_{2}\;dW_{2}(t))\\ \;+k_{1,2}\;(q_{1}(t)\;dt+\sigma_{1}\;dW_{1}(t)), \end{array}$$

$$\begin{array}{c} \;dq_{3}(t)=\;\;\;\;\;-(k_{1,3}+k_{4,3})\;(q_{3}(t)\;dt+\sigma_{3}\;dW_{3}(t))\\ \;+0.1k_{4,3}\;(q_{4}(t)\;dt+\sigma_{4}\;dW_{4}(t))\\ \;+k_{3,1}\;(q_{1}(t)\;dt+\sigma_{1}\;dW_{1}(t)),\\ dq_{4}(t)=\;\;\;\;-0.1k_{4,3}\;(q_{4}(t)\;dt+\sigma_{4}\;dW_{4}(t))\\ \;+k_{4,3}\;(q_{3}(t)\;dt+\sigma_{3}\;dW_{3}(t)). \end{array}$$

We fix the diffusion parameters to be *σ*_1_=*σ*_2_=*σ*_3_=*σ*_4_=3. Initial conditions are given by

$$q_{1}(0)=q_{2}(0)=q_{3}(0)=q_{4}(0)=0.$$

The patients get a bolus injection and therefore the input *u*_1_(*t*) will be modelled as a delta distribution at time *t*=0h,

$$u_{1}(t)=u_{1,0}\delta (t).$$

Practically, this means that only the initial condition *q*_1_ is affected by it, and we can replace the initial condition for *q*_1_ by

$$q_{1}(0)=u_{1,0},$$

and set *u*_1_(*t*)=0 in the differential equations.

The same differential equations, without noise terms, are assumed to be valid for the states *Q*_*i*_ and the input *U*_1_ of the tracee:

$$\frac{dQ(t)}{dt}=K(t)Q(t)+U(t)$$

where $Q(t)={\left(Q_{i}(t)\right)}_{i=1,2,3,4}^{T}$

, *u*(*t*)=(*u*_1_(*t*),0,0,0)^T^. It is assumed that the tracee input *U*_1_(*t*)=*U*_1_ is constant but unknown. We will therefore estimate it together with the transfer parameters. Since for the tracee steady state is assumed (i.e. d*Q*_*i*_(*t*)/ d*t*=0), it is possible to solve those equations for *Q*_1_(*t*), and we get:

$$Q_{1}(t)=\frac{(k_{11,2}+k_{1,2})U_{1}}{k_{0,1}(k_{11,2}+k_{1,2})+k_{11,2}k_{1,2}}.$$

The output is a measurement proportional to the ratio between the tracer and the tracee, disturbed by log-normal noise:

$$\begin{array}{cc} y_{1}(t)=p_{1}\frac{q_{1}(t)}{Q_{1}(t)}\xi_{t},\;\xi_{t}∼ & \text{Log-}N\left(0,\sigma_{y_{1}}^{2}\right)\text{independently}\\ \;\text{for each}\;\;t, \end{array}$$

where we assume the value of the variance parameter to be $\sigma_{y_{1}}^{2}=0.5^{2}$ (this is the variance of log*ξ*_*t*_). The parameter *p*_1_ denotes the unknown proportion of plasma leucine that actually is in the plasma. The parameters *p*_1_ and *U*_1_ are not jointly identifiable, therefore we fix *p*_1_=0.65. More details concerning the deterministic model (without noise terms) can be found in [31] and [33]. Note that the stochastic disturbances are not part of the original model, they are rather augmentations of the model used in this article.

#### The mixed effects model

The model as presented in the last paragraph contains only flux parameters *k*_*j*,*i*_ which are assumed to be the same for every individual. Neither does it account for individual differences between several persons, nor does it account for possible changes of the flux parameters when the persons under consideration are affected by a disease or a treatment. To be able to treat these differences in an appropriate way, we introduce group and patient specific parameters in the model; namely the transfer coefficient *k*_0,1_ will be split into a group dependent and a patient dependent part. In this way, we introduce so-called mixed effects into the model. Mixed effects generally increase the difficulty in inference making. In the following test runs, we will use measurement data previously reported as individual data for a total of 34 subjects [31,32]. Among them, 15 patients belonged to the diabetes group, and 19 to the control group. From experiments, it can be observed that the degradation rate *k*_0,1_ of plasma leucine is significantly different for people with and without diabetes. We therefore assume that the expected value of *k*_0,1_ in each group differs and may be either $k_{0,1}^{d}$ or $k_{0,1}^{c}$ corresponding to the diabetes group and the control group, respectively. Additionally, we assume that we have patient-dependent random factors *ζ*_*p*_ modelling the parametric uncertainties among individuals, such that finally

$$k_{0,1}^{\left(p\right)}=\left\{\begin{array}{c} \zeta_{p}k_{0,1}^{d}\;\text{if the patient}\;p\;\text{is in the diabetes group},\\ \zeta_{p}k_{0,1}^{c}\;\text{if the patient}\;p\;\text{is in the control group} \end{array}\right)$$

where all *ζ*_*p*_’s are static and independently log-normally distributed:

$$\begin{array}{cc} \zeta_{p}=\exp(\eta_{p})\; & \text{with}\;\;\eta_{p}∼N\left(0,\sigma_{\eta_{p}}^{2}\right)\\ \;\text{independently for each}\;\;p \end{array}$$

for *p* = 1,…,34. As a consequence, each of the states *q*_1_,…,*q*_4_ has to be considered separately for each patient *p*. We indicate this by writing $q_{1}^{\left(p\right)},\ldots ,q_{4}^{\left(p\right)}$, *p*=1,…,34.

The aim of the estimation runs is, apart from estimating the remaining parameters, to show that the group dependent parameters $k_{0,1}^{d}$, $k_{0,1}^{c}$ are indeed different. We want to apply Bayesian estimation to the parameters. For this reason, we principally have to treat them like state variables in the particle filter. Since the estimation of static variables is problematic with particle filter methods, it is standard to introduce small artificial stochastic dynamics to the parameters consisting of normal increments with decaying variances [35]. The same is true for the static noise parameters *η*_*p*_ which also are to be estimated. Our process *X*_*t*_ is then given as an augmented state vector

$$\begin{array}{l} X_{t}=\left(q_{1:4}^{\left(1:34\right)}(t),k_{0,1}^{c}(t),k_{0,1}^{\;d}(t),k_{1,2}(t),k_{1,3}(t),k_{3,1}(t),\right)\;\\ \;{\left(k_{4,3}(t),U_{1}(t),\eta_{1:34}(t)\right)}^{T}.\; \end{array}$$

The overall model is thus a non-linear mixed effects model with three levels of effects (parameters), namely global parameters, group dependent parameters ($k_{0,1}^{d}$, $k_{0,1}^{c}$), and personal parameters (*ζ*_*p*_). Nevertheless, since the core of the model is linear, i.e. the states *q*_1_,…,*q*_4_ conditioned on all parameters, a Rao-Blackwellization concerning the linear parts of the model is possible, and the Kalman filter applied to this linear partial model can be used in combination with particle filtering for the non-linear parts [36]. Since the model is used for demonstration purposes only, we did not use this technique although in principle it is possible.

#### Estimation runs

Estimation and simulation runs have been performed with data from all 34 patients (19 from control group and 15 from diabetes group). The computer experiments have been done as follows. We first estimate parameters with the MTU particle filter. Separately, we estimate parameters with the standard particle filter under the same conditions and with the same seed for the random number generator. The initial distribution of the particles is then the same in both cases. For both runs, we compute an estimate of the effective sample size (ESS) and the data likelihood over time. Both estimates allow a performance comparison of the MTU versus the standard particle filter. Secondly, the empirical medians of the final parameter distributions are afterwards used to perform simulation runs in both cases. Both versions of the particle filter, this time with parameters fixed to the estimated values, are used to perform these simulations. In these simulation runs, the data are used for the computation of the data likelihood conditioned on the estimated parameters. The resulting distributions of the simulated measurements can be compared to the true measurements, both visually and by observing the data likelihood.

We used 10,000 particles and a resampling threshold of 7,500. Stepsizes in the MTU filter are between 10^-7^h and 10^-3^h, adaptively computed based on the ESS estimate. In the standard filter, we use a fixed stepsize of 10^-3^h. The data contain measurements until time *t*=8h, but we perform estimations and simulations only until time *t*=1h, mainly to reduce computation time; anyhow, after *t*=1h, the tracer concentrations are relatively low and do not change considerably, and therefore the measurements are not expected to improve the estimations significantly. In our implementation, we directly sample from the states *X*_*t*_ (i.e. ${\tilde{X}}_{\left[t_{0},\infty \right)}=X_{\left[t_{0},\infty \right)}$ in law) using the Euler-Maruyama scheme for discretization [37].

As mentioned earlier, Bayesian parameter estimation with particle filters requires the introduction of artificial dynamic noise for the parameters. It is standard to use normal increments with decaying variances [35]. Since in our case all parameters with exception of the *η*_*p*_’s are assumed to be positive, the application of a “log-normal” noise (based on a geometric Brownian motion) in place of the standard normal noise seems to be more appropriate for these parameters. In detail, it has been done as follows. The priors and the distributions of the artificial noise are chosen to be log-normal for all parameters with exception of the individual parameters *η*_*p*_ which have normal priors and noise. The prior for the parameters $k_{0,1}^{c}$, $k_{0,1}^{d}$, *k*_1,2_, *k*_1,3_, *k*_3,1_, *k*_4,3_ is $\text{Log}-N(0,1^{2})$, the prior for *U*_1_ is $\text{Log}-N(\log(100),1^{2})$. The prior for each *η*_*p*_ is $N(0,0.5^{2})$. The parameter update (“artificial noise”) is generally performed according to

$$d\theta (t)=\theta (t)\sigma_{\theta }(t)\;dW_{t}^{\theta }$$

for $\theta =k_{0,1}^{c}$, $k_{0,1}^{d}$, *k*_1,2_, *k*_1,3_, *k*_3,1_, *k*_4,3_, *U*_1_, and according to

$$d\theta (t)=\sigma_{\theta }(t)\;dW_{t}^{\theta }$$

for *θ*=*η*_*p*_. It is standard to decrease the variance of the artificial noise over time. In our case we have chosen the diffusion parameter *σ*_*θ*_ of each artificial noise variable to be dependent on time via a quadratic function

$$\sigma_{\theta }(t)=a_{\theta }/{\left(t-b_{\theta }\right)}^{2}$$

with parameter dependent coefficients *a*_*θ*_ and *b*_*θ*_. Practically, *a*_*θ*_ and *b*_*θ*_ have been determined by fixing two interpolation points (*t*_0_,*σ*_*θ*_(*t*_0_)) and (*t*_1_,*σ*_*θ*_(*t*_1_)). It holds:

$$\begin{array}{lll} b_{\theta } & =(t_{1}-t_{0})/\left(1-\sqrt{\sigma_{\theta }(t_{0})/\sigma_{\theta }(t_{1})}\right)\;\text{and}\;\; & \;\\ a_{\theta } & =\sigma_{\theta }(t_{0}){\left(t_{0}-b_{\theta }\right)}^{2}.\; & \; \end{array}$$

We found best performance with the following choices: For all parameters, we have chosen *t*_0_=0h, *t*_1_=2h and *σ*_*θ*_(*t*_1_)=*σ*_*θ*_(*t*_0_)/10 which means that the diffusion parameter has dropped to 10% of its initial value at time *t*=2h. The initial values are *σ*_*θ*_(*t*_0_)=0.5h for all parameters with exception of the *η*_*p*_’s which have higher initial diffusion *σ*_*θ*_(*t*_0_)=1h.

**Figure 9:**
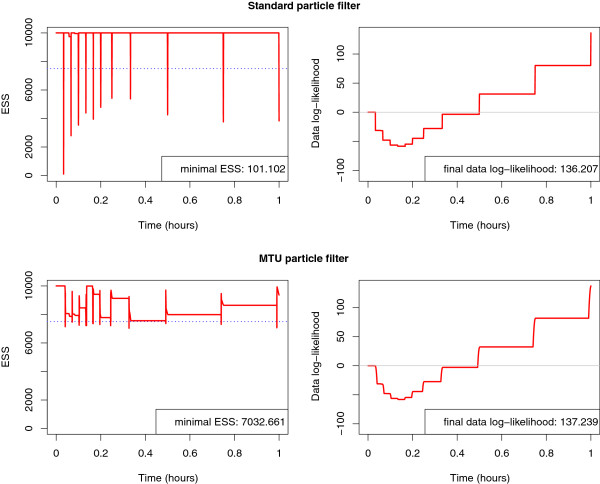
**Development of estimated effective sample size and estimated data likelihood during estimation runs.** Standard particle filter (top) and MTU particle filter (bottom).

**Figure 10:**
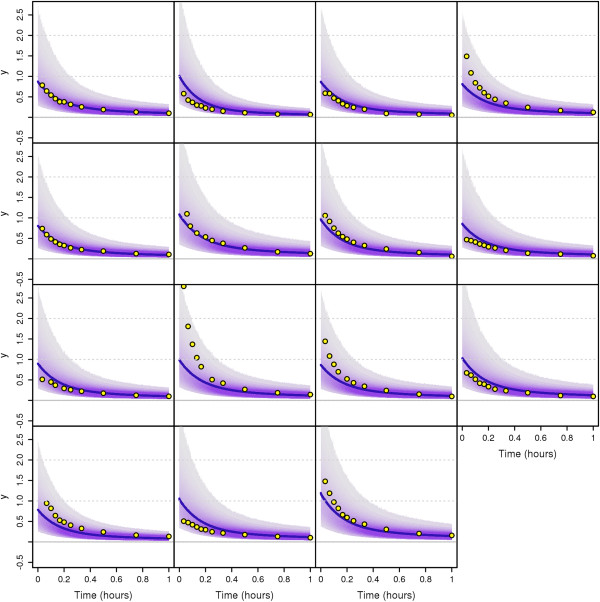
**Predicted measurement distribution over time (standard particle filter) during simulation run. Patients from diabetes group.** Development of predicted measurement distributions over time during simulation run with parameters estimated by the standard particle filter. Circles: Measurements. Solid line: Median of simulated measurements. Violet shaded area: Measurement distributions based on empirical quantiles.

**Figure 11:**
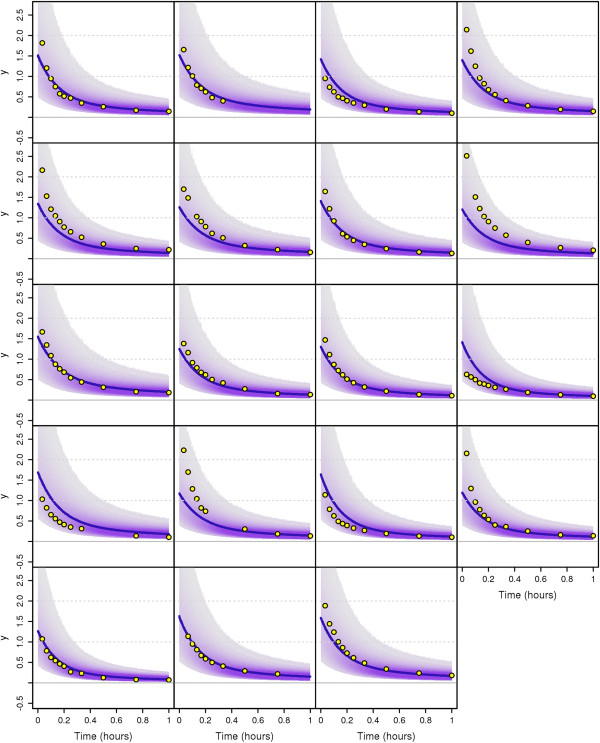
**Predicted measurement distribution over time (standard particle filter) during simulation run. Patients from control group.** Development of predicted measurement distributions over time during simulation run with parameters estimated by the standard particle filter. Circles: Measurements. Solid line: Median of simulated measurements. Violet shaded area: Measurement distributions based on empirical quantiles.

**Figure 12:**
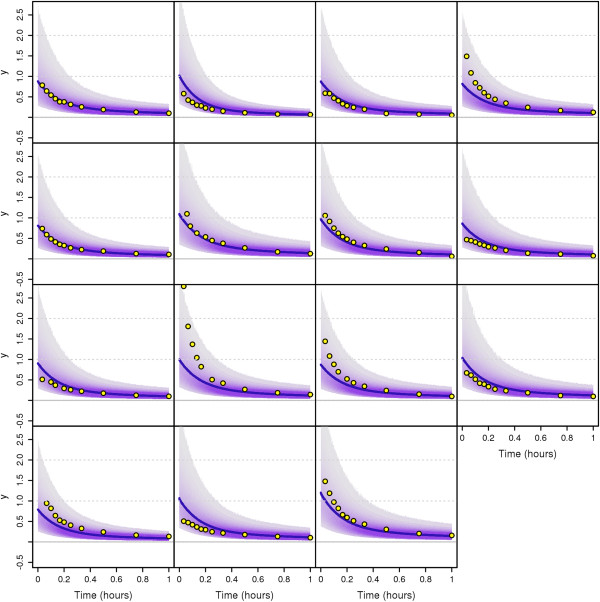
**Predicted measurement distribution over time (MTU particle filter) during simulation run. Patients from diabetes group.** Development of predicted measurement distributions over time during simulation run with parameters estimated by the MTU particle filter. Circles: Measurements. Solid line: Median of simulated measurements. Violet shaded area: Measurement distributions based on empirical quantiles.

**Figure 13:**
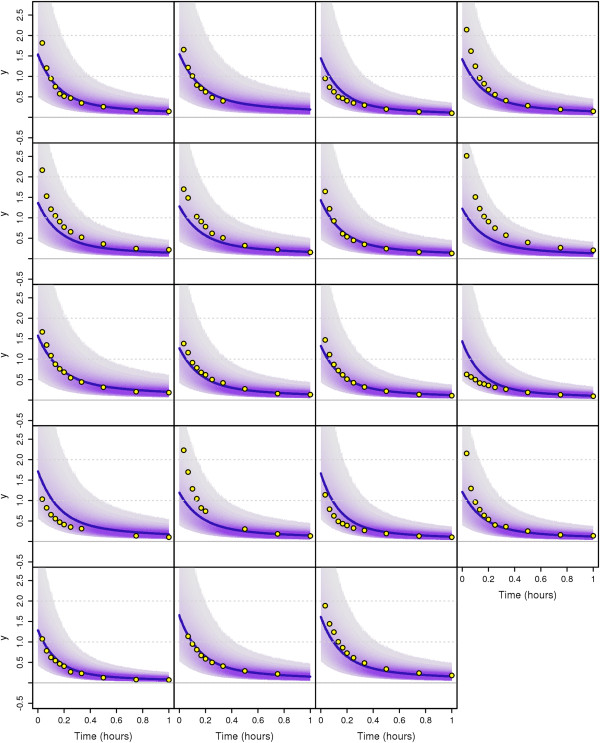
**Predicted measurement distribution over time (MTU particle filter) during simulation run. Patients from control group.** Development of predicted measurement distributions over time during simulation run with parameters estimated by the MTU particle filter. Circles: Measurements. Solid line: Median of simulated measurements. Violet shaded area: Measurement distributions based on empirical quantiles.

**Figure 14:**
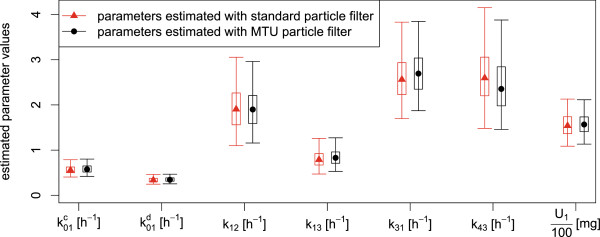
**Estimated global and group parameters.** Box plots of estimated distributions for the global parameters and the group dependent parameters. The medians are depicted with triangles (standard particle filter) or circles (MTU particle filter). The bottom and top of the box are the 0.25-quantile and the 0.75-quantile, i.e. 50% of the values lie within the box. The whiskers mark the 0.025-quantile and the 0.975-quantile, i.e. 95% of the values lie between the whiskers. The values for *U*_1_ have been scaled by a factor of 0.01 in order to fit into the plot.

**Figure 15:**
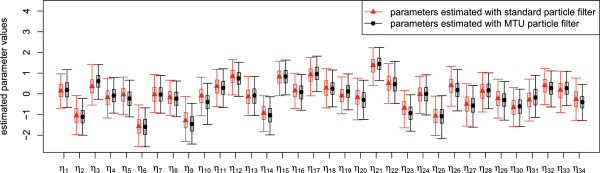
**Estimated individual parameters.** Box plots of estimated distributions for the individual parameters. The medians are depicted with triangles (standard particle filter) or circles (MTU particle filter). The bottom and top of the box are the 0.25-quantile and the 0.75-quantile, i.e. 50% of the values lie within the box. The whiskers mark the 0.025-quantile and the 0.975-quantile, i.e. 95% of the values lie between the whiskers.

As mentioned earlier, we have performed two different estimation and simulation runs, one with the MTU particle filter with distributed measurement times, and for comparison one run with the standard particle filter. In the MTU particle filter, the distributions of the measurement times are truncated normal distributions with mean equal to the nominal value of the measurement time and with variance 0.001^2^. The normal distribution is truncated at the time point 0.01h left and right from the mean value. In Figure 9, the development of the estimated effective sample size and the estimated data likelihood is shown, both with respect to time *t*. The development of predictions of the measurements during a simulation run with the final estimated parameters is shown in Figures 10 and 11 for the run with the standard particle filter, and in Figures 12 and 13 for the run with the MTU particle filter. Finally, in Figures 14 and 15, box plots of the final estimated global and individual parameters are shown, respectively.

A comparison of the results of MTU-PF and standard particle filter shows that both algorithms exhibit very similar performance with respect to the quality of the estimated parameters, since in both cases the development of the data likelihood is very similar both for estimation and simulation. The estimated log-likelihood of the data at the final time is 137.239 for the MTU particle filter and 136.207 in the standard case, which is practically equal. The computation time of the MTU-PF is only slightly higher than the one of the standard filter. Visual inspection of the simulation runs shows that the simulated measurements of the model with parameters estimated by both filters fit the data in a similar way. This impression is supported by the values of the estimated data likelihood. The MTU particle filter has a final log-likelihood value of 157.622, similar to the one of the standard filter with a value of 155.952. The difference is insignificant.

In contrast to the insignificant differences concerning the resulting likelihoods between the MTU-PF and the standard PF, the development of the ESS estimate in the estimation runs differs remarkably. With the MTU particle filter, the ESS estimate shows a high value at all times and does not drop below a value of 7032.661 during estimation. This is only slightly lower than the resampling threshold of 7500 (see Figure 9, upper part). The standard particle filter shows a much worse performance. As can be observed from the lower part of Figure 9, the ESS drops several times to very low values, reaching a minimum of 101.102. The ESS measures in some sense the variance of the normalized particle weights (low ESS means high variance) and highly varying weights indicate that the particle cloud is in a bad condition, since in this case a few particles with very high weights dominate the majority of the particles with very low weights. The extreme case is ESS=1 where only one particle has a significant weight. After a resampling step, only the information carried by this particle is present in the current particle cloud. Results obtained by estimation runs where such low ESS values have occurred cannot be trusted very much. If in contrast a resampling step is performed while the ESS is only slightly below the resampling threshold, which is the case in the MTU-PF algorithm, then many different particles will be chosen during resampling and a high percentage of the information contained in the particle cloud will be carried over to subsequent steps. In this sense, the MTU particle filter avoids the degeneration of the particle cloud by controlling the value of the ESS and holding it at a high value at every time. As a consequence, the estimation results should be considered to be more reliable than with the standard algorithm.

A look at the estimated values of the group parameters $k_{0,1}^{c}$ and $k_{0,1}^{d}$ (see Figure 14) shows that in both estimation runs (MTU-PF and standard PF), the rate $k_{0,1}^{d}$ for diabetes patients is only about 60 percent of the rate $k_{0,1}^{c}$ of the control group (standard particle filter: 0.337h^-1^ vs 0.557h^-1^, MTU particle filter: 0.346h^-1^ vs 0.577h^-1^). The good performance especially of the MTU particle filter strengthens the confidence in the obtained result and leads to the conclusion that the secretion rate *k*_0,1_ is indeed lower for the diabetes patients than for the people from the control group.

## Conclusions and future work

We proposed a new modification of the particle filter algorithm which works in continuous-time settings. It allows the direct inclusion of measurement time uncertainties in the underlying model. The modifications additionally allow the use of time-stepping strategies to improve the performance of the algorithm. The assumption of a random distribution of measurement times is natural in many applications.

The MTU-PF method is generally applicable. Even when measurement times may be assumed to be concentrated on single time points, our method can be used as a kind of regularization of the standard particle filter method if artificial distributions with highly concentrated masses around the measurement points are introduced.

We compared the performance of the MTU-PF to the standard PF and to an alternative Maximum Likelihood estimation method on a small artificial example. The results clearly show the advantage of the application of the MTU-PF in cases of uncertain measurement times.

We believe that our MTU particle filter is especially suitable for biological/medical applications where — compared to technical applications — the variance of the measurement values is relatively high due to biological variation and because relatively few consecutive measurements are possible. We provided an illustrative application from a PK/PD study. A comparison of our MTU particle filter with the standard filter showed that in this case our method is able to avoid weight degeneracy measured in terms of the Effective Sample Size (ESS) estimate. Even though the estimations in this application with both standard and MTU particle filters are reasonably good, the fit to the measurements is still not perfect. Whether that is due to our choices of model and parameters, or due to the known weaknesses of the general SMC method (which also our modification necessarily suffers from), remains to be evaluated in greater detail. In subsequent studies, we plan to apply the new algorithm to the complete liver-plasma model using additional measurements to be able to draw conclusions of greater medical relevance.

Another topic may also prove interesting for future work. Our experiments showed that the results are highly dependent on the choice of the development of the diffusion coefficients of the artificial noise necessary for Bayesian parameter estimation. While there is a general agreement that these coefficients should decrease over time, there is currently a lack of automated methods for making appropriate choices of the diffusion coefficients, both for initial values and dynamic development. However, to provide a really practical method for parameter estimation in non-linear mixed effects models (or even in models which adhibit only global parameters), our approach could also be combined with methods better suited to the estimation of fixed parameters, a good candidate being the PMCMC methods proposed in [11]. This is future work.

In summary, we believe that the method presented in this article opens the door to even more efficient and reliable state sampling and parameter estimation methods based on the particle filter algorithm operating on continuous-time stochastic state space systems.

## Notation

(Ω,A,P) probability space

$\left(X_{t},B_{X_{t}}\right)$ arbitrary measurable space (for each *t*∈[*t*_0_,*∞*) with $t_{0}\in R$) 

$X_{t}:\Omega \to X_{t}$$A-B_{X_{t}}$ measurable random variable 

$X_{\left[t_{0},\infty \right)}:\;\;=\;{\left(X_{t}\right)}_{t\in [t_{0},\infty )}$ continuous-time Markov process with general state space $X_{\left[t_{0},\infty \right)}$

$X_{\left[t_{0},\infty \right)}:\;=\underset{t_{0}\leq s}{\prod }X_{s}$ state space of $X_{\left[t_{0},\infty \right)}$

$L_{X_{t}}$ the pushforward measure of P under *X*_*t*_, i.e. $L_{X_{t}}(B):\;=$$\text{P}(X_{t}^{-1}(B))$ for all $B\in B_{X_{t}}$

$L_{X_{\left[t_{0},\infty \right)}}$ the pushforward measure of P under $X_{\left[t_{0},\infty \right)}:\;={\left(X_{s}\right)}_{s\in [t_{0},\infty )}$ (with the corresponding product algebra) 

$X_{\left[t_{0},t\right]}:\;=\underset{t_{0}\leq s\leq t}{\prod }X_{s}$ the state space restricted to the interval [*t*_0_,*t*]

$L_{X_{\left[t_{0},t\right]}}$ the corresponding pushforward measure

*N* number of particles 

${\left(x_{t}^{i}\right)}_{i=1,\ldots ,N}$ state samples at time *t*

*K*_*s*,*t*_(*x*_*s*_, d*x*_*t*_) the Markov kernel of the process $X_{\left[t_{0},\infty \right)}$ from time *s* to time *t*

*a*(*x*,*t*) drift vector 

*B*(*x*,*t*) diffusion matrix 

$W_{t}$ multidimensional standard Wiener process 

$\left(Y_{j},B_{Y_{j}}\right)$ measurable space 

*Y*_1:*M*_ observation random variable with values in measurable spaces $\left(Y_{j},B_{Y_{j}}\right)$

$g_{j}(y_{j}\;|\;x_{t_{j}},t_{j})$ conditional probability density with respect to a reference measure $\mu_{Y_{j}}$

$\mu_{Y_{j}}$ reference measure $\mu_{Y_{j}}$ on$\left(Y_{j},B_{Y_{j}}\right)$

*t*_*j*_ (*j*=1,…,*M*) observation times 

*T*_*j*_ random variables modelling the uncertainty about exact observation times 

$\lambda_{\left[t_{0},\infty \right)}$ Lebesgue measure on the interval [*t*_0_,*∞*) 

*γ*_*j*_(*t*_*j*_) probability density of *T*_*j*_ with respect to $\lambda_{\left[t_{0},\infty \right)}$

${\tilde{X}}_{t_{0:M}}$, $L_{{\tilde{X}}_{t_{j}}}$, and Markov chain, pushforward

${\tilde{K}}_{t_{j-1},t_{j}}(x_{t_{j-1}},\;dx_{t_{j}})$ measure, and kernel for importance sampling 

$\varrho_{t_{j}\;|\;t_{j-1}}(x_{t_{j}}\;|\;x_{t_{j-1}})$ Radon-Nikodym derivative$\frac{K_{t_{j-1},t_{j}}(x_{t_{j-1}},\;dx_{t_{j}})}{{\tilde{K}}_{t_{j-1},t_{j}}(x_{t_{j-1}},\;dx_{t_{j}})}$

$\varrho_{t_{0}}(x_{t_{0}}):\;=\frac{\;dL_{X_{t_{0}}}(x_{t_{0}})}{\;dL_{{\tilde{X}}_{t_{0}}}(x_{t_{0}})}$ Radon-Nikodym derivative at start time *t*_0_

$w_{t}^{i}$ unnormalized weight of particle *i* at time *t*

${\tilde{w}}_{t}^{i}:\;=\frac{w_{t}^{i}}{\overset{N}{\underset{\nu =1}{\sum }}w_{t}^{\nu }}$ normalized weight of particle *i* at time *t*

*s*_*ℓ*_*ℓ*-th resampling time 

$v_{s_{\ell }}^{i}$ (unnormalized) selection weight 

$p_{\ell }^{i}$ normalized selection weight (probability that particle *i* will be selected during resampling) 

*ι*_*ℓ*_:*I*→*I* selection function on the index set *I*: ={1,…,*N*} for the *ℓ*-th resampling step 

$Z_{t_{k}}(t_{1:M})$ data likelihood 

*f*_*t*_ full density at time *t*

${\hat{f}}_{t}$ filter density at time *t*, i.e. only those observations *y*_*j*_ are included for which *t*_*j*_≤*t*

${\bar{g}}_{j,t}(y_{j}\;|\;x_{t_{j}},t_{j})$ conditional probability density $g_{j}(y_{j}\;|\;x_{t_{j}},t_{j})$ if *T*_*j*_≤*t*, 1 otherwise 

$W_{j,t}:\Omega \to R_{\geq 0}$ stochastic process given by d*W*_*j*,*t*_(*ω*)=(*g*_*j*_(*y*_*j*_ | *X*_*t*_(*ω*),*t*) -1)*γ*_*j*_(*t*) d*t*

$W_{t}:\Omega \to R_{\geq 0}$ product process of the *W*_*j*,*t*_, where *j*=1,…,*M*

${\hat{E}}_{t}[h(X_{t})\;|\;Y_{1:M}=y_{1:M}]$ expectation of *h*(*X*_*t*_) given *Y*_1:*M*_=*y*_1:*M*_ with respect to the filtered state *X*_*t*_

$w_{j,t}:X_{\left[t_{0},t\right]}(\Omega )\to R_{\geq 0}$ partial weight at time *t* (w.r.t. the *j*-th observation) 

$w_{t}:X_{\left[t_{0},t\right]}(\Omega )\to R_{\geq 0}$ weight at time *t*

${\bar{\gamma }}_{j,t}$ cumulative distribution function $\int_{t_{0}}^{t}\gamma_{j}(t_{j})\;dt_{j}$

${\bar{w}}_{j,t}(x_{\left[t_{0},t\right]})$$\int_{t_{0}}^{t}g_{j}(y_{j}\;|\;x_{t_{j}},t_{j})\gamma_{j}(t_{j})\;dt_{j}$

*t*_0_=*τ*_0_<*τ*_1_…<*τ*_*D*_ time discretization 

${\bar{v}}^{i}$ cumulative product of the selection weights for particle *i*

*Δτ*_*d*_=*τ*_*d*_-*τ*_*d*-1_ stepsize 

${\bar{Z}}_{\tau_{d}}$ correction factor for the data likelihood at time *τ*_*d*_

*Q*_*i*_ mass of the tracee in compartment *i*

*q*_*i*_ mass of the tracer in compartment *i*

*U*_*i*_ input for the tracee in compartment *i* (e.g. *U*_1_ denotes the influx into compartment 1) 

*u*_*i*_ input for the tracer in compartment *i*

*k*_*j*,*i*_ transfer coefficient of the tracers from compartment *i* to compartment *j*

*σ*_*i*_ diffusion parameter 

*ξ*_*t*_ log-normal noise ($\xi_{t}∼\text{Log-}N(0,\sigma_{y_{1}}^{2})$) 

$k_{0,1}^{d}$, $k_{0,1}^{c}$ degradation rate of plasma leucine for people in the diabetes group and the control group, respectively 

*ζ*_*p*_, *η*_*p*_ patient-dependent random factors modelling the parametric uncertainties among individuals (*ζ*_*p*_= exp(*η*_*p*_) with $\eta_{p}∼N(0,\sigma_{\eta_{p}}^{2})$)

## Competing interests

The authors declare that they have no competing interests.

## Supplementary Material

Additional file 1 **Data used for estimation and simulation.** The data consist of one record for each patient, each record consisting of 4 lines in the following format:
patient id [COMMA] group (control or diabetes) [COMMA] leucine initial value [NEW LINE]measurement 1 time [COMMA] measurement 2 time [COMMA] …[COMMA] measurement *n*_*p*_ time [NEW LINE]measurement 1 value[COMMA] measurement 2 value [COMMA] …[COMMA] measurement *n*_*p*_ value [NEW LINE][NEW LINE]
where *n*_*p*_ is the number of measurements for patient *p*, time is given in hours, and each measurement value is the measured ratio of isotope labeled leucine. Click here for file
